# Brain–Behavior Relationships in Children With ADHD: Resting‐state EEG Networks Underlying Executive Function and Neurological Soft Signs

**DOI:** 10.1002/brb3.71698

**Published:** 2026-08-20

**Authors:** Merve Kuz, Halit Necmi Uçar, Serap Aydın, Fatih Hilmi Çetin, Serhat Türkoğlu

**Affiliations:** ^1^ Diamind Mental Academy Child‐Adolescent Psychiatry & Psychotherapy Clinic Konya Türkiye; ^2^ Medical Faculty Biophysics Department Hacettepe University Ankara Türkiye; ^3^ Department of Child and Adolescent Psychiatry Selçuk University Konya Türkiye

**Keywords:** ADHD, brain network, neurological soft signs, resting‐state EEG, stroop test battery

## Abstract

**Purpose:**

To investigate sex‐stratified brain–behavior relationships between large‐scale functional brain network topology and executive‐motor profiles in pediatric ADHD, using a multi‐dimensional parameter exploration approach.

**Method:**

Graph‐theoretical connectivity measures were derived from resting‐state EEG in 41 medication‐naïve children with ADHD (10 girls) and 42 healthy controls (HCs) (10 girls), aged 6–12 years. Sex‐specific within‐ and between‐group differences were examined across temporal windows (1‐2‐4 s), frequency bands, and network densities (10%–30%). Associations between connectivity measures and behavioral/clinical profiles (Stroop, PANESS, Turgay DSM‐IV scales) were also assessed.

**Findings:**

Directed Transfer Function showed more reliable predictions than phase‐sensitive estimators. ADHD‐girls showed lower local connectivity, and HC‐girls showed higher integration/segregation in within‐group gender differences in lower alpha at sparse network densities (10%–15%). Network alterations and meaningful brain‐behavioral relationships were limited to upper alpha and beta at denser network configurations (20%–30%) in ADHD‐girls. Total gait/station was negatively correlated with integration in HC, positively correlated with hub‐to‐hub organization in ADHD. The edge‐level differences were localized at right centro‐temporal locations (FC6‐T8) in boys, while the fewer and weaker differences were localized at antero‐posterior, occipital‐centric connections converging onto AF3 locus in girls.

**Conclusion:**

The density‐dependent emergence of brain–behavior correlations in girls offers a plausible network‐level explanation for the diagnostic camouflage effect reported in females with ADHD. Pediatric ADHD manifests through sex‐specific network reconfigurations, a right‐hemispheric inhibitory‐circuit deficit in boys versus an antero‐posterior sensory‐gating alteration in girls.

## Graphical abstract

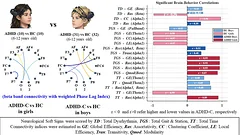

## 1 Introduction

Attention‐deficit/hyperactivity disorder (ADHD) is a neurodevelopmental condition characterized by persistent impairments in attention, inhibitory control, and behavioral regulation, with onset in childhood and substantial heterogeneity in clinical presentation (American Psychiatric Association 2013). Within systems‐level framework, executive dysfunction, typically indexed by impairments in inhibitory control, working memory, and attentional regulation, has been extensively investigated and is considered a core feature of ADHD (Willcutt et al. 2005). Contemporary neurocognitive models conceptualize ADHD not as a focal deficit but as a disorder of distributed brain systems, particularly involving dysregulated interactions across fronto‐striatal, fronto‐parietal, and cerebellar networks that support executive control and goal‐directed behavior (Nigg and Casey 2005; Konrad and Eickhoff 2010; Posner et al. 2014).

In the past, the results of a study estimating the local power distributions of resting‐state surface EEG recordings in children diagnosed with ADHD combined type and ADHD predominantly inattentive type supported the idea that in two‐component pediatric ADHD symptoms, the hyperactive/impulsive component matures with age, while the attention deficit component remains more stable (Clarke et al. 2001). These results point to age‐related changes in brain activity and gender‐dependent neurodevelopmental differences in ADHD. According to a later EEG local power distribution analysis conducted by the same researchers on only girls and a matched control group, it has been noted that in female patients, a pattern of rapid band suppression with slow activity increase was observed; that they differed most significantly from controls in the frontal regions; and that there was heterogeneity among patients (much higher theta, lower alpha/beta, higher total power), that is, neurophysiological subtypes (Clarke et al. 2003).

For many years, the most popular EEG findings for ADHD were spectral findings (high theta, low beta, and high theta/beta ratio), but over the years it was noticed that these became inconsistent with age and ADHD subtypes. Therefore, in a study testing the diagnostic discrimination of EEG patterns in child and adult ADHD groups, it was shown that in childhood, both the patient and control groups shared the known pattern of normal maturation (slow waves decrease and faster rhythms become dominant), but children with ADHD, characterized by higher theta activity, had a more immature electrophysiology of the brain (Poil et al. 2014). These findings are consistent with the developmental delay hypothesis. However, the question of whether the non‐normalized network dynamics observed in adult ADHD are due to developmental delay or a permanent reorganization is open to debate.

Today, neuroscientific research on ADHD is increasingly focusing on functional and structural connectivity analyses. Connectivity studies based on resting‐state EEG analyses show findings of a more centralized functional network in children with ADHD (33 boys, 9 girls, and 7–14 years) compared to typically developing children (32 boys and 11 girls) (Janssen et al. 2017). A structural connectivity analysis study based on MRI data, conducted only between ADHD‐combined type boys and typically developing healthy boys, points to delayed brain maturation and impaired network organization in ADHD boys (Beare et al. 2017). A resting‐state fMRI study indicated that children with ADHD (89 boys and 30 girls, ages 7–14) showed less integrated topology and more dispersed/random organization of the frontoparietal attention network, which is critical for attention and executive control (Y. Wang et al. 2019). The researchers also investigated how resting brain signals form temporally repeating rhythms/cycles and showed disruptions in temporal synchronization patterns in patient groups (ASD and ADHD) based on resting‐state fMRI (Y. Wang et al. 2020). These findings suggest that ADHD may involve abnormalities not only in regional activation levels but also in the temporal coordination dynamics of intrinsic brain networks. In an animal experiment where Wistar Kyoto rats served as the control group, spontaneously hypertensive rats exhibiting ADHD‐like hyperactivity and impulsivity showed delayed brain network maturation as a result of “metabolic connectivity” analysis using FDG‐PET (Ha et al. 2020).

According to Barkley, the core disorder in ADHD is an inhibition problem. That is, the individual has difficulty stopping dominant impulses, delaying responses, and suppressing distractions. This inhibition disorder impairs higher‐level executive functions (Barkley 2012). In subsequent years, it has been argued that ADHD may not be a problem of “information deficiency” but rather a dysfunction of the self‐regulation systems that enable the individual to organize their behavior goal‐oriented over time (Antshel et al. 2014). Considering the findings of connectivity analyses (such as impaired frontoparietal network organization, low network integration, developmental maturation delay, and atypical DMN regulation), viewing ADHD solely as an attention problem is insufficient; the main issue may be the dysfunction of executive control and self‐regulation systems that organize the individual's behavior over time. Therefore, ADHD should be understood as a cognitive, temporal, motivational, and behavioral self‐management disorder. If ADHD reflects atypical maturation and organization of distributed control networks, such dysfunction may extend beyond executive and attentional systems to motor and sensorimotor domains that depend on coordinated integration across cortical, subcortical, and cerebellar circuits. Within this framework, neurological soft signs (NSS) may represent a behavioral manifestation of impaired network maturation and sensorimotor integration rather than isolated motor abnormalities (Denckla 1985; D'agati et al. 2018). Consistent with this view, NSS are frequently observed in children with ADHD and have been linked to atypical development of distributed motor and cerebellar circuits (Mostofsky et al. 2003; Mostofsky et al. 2006; Mostofsky et al. 2007). Evidence from other neuropsychiatric conditions further suggests that NSS may reflect broader disturbances in large‐scale brain organization rather than isolated motor abnormalities. Supporting this view, findings from other neuropsychiatric conditions suggest that elevated NSS levels are associated not only with motor abnormalities but also with broader cognitive dysfunction. On the other hand, no significant relationship was found between the severity of OCD symptoms and treatment resistance and NSS scores (Doolub et al. 2024). From this perspective, executive dysfunction and NSS may represent partially distinct behavioral expressions of a shared disruption in large‐scale network maturation and integration.

Network neuroscience provides a formal framework to quantify such distributed organization. Graph theoretical approaches model the brain as a complex network in which nodes represent neural elements and edges represent functional interactions. Within this framework, global efficiency (GE) indexes the capacity for distributed information integration, whereas clustering coefficient (CC) and local efficiency (LE) reflect the degree of specialized, local processing. Modularity (Q) captures the extent to which the network is partitioned into relatively segregated submodules (Rubinov and Sporns 2010; Bullmore and Sporns 2012). Efficient cognitive function is thought to depend on an optimal balance between integration and segregation. Deviations from this balance have been reported across neurodevelopmental disorders, including ADHD, where studies indicate reduced GE alongside altered modular organization, suggesting a shift toward less integrated and more segregated network configurations (L. Wang et al. 2009; Lin et al. 2014; Beare et al. 2017).

Electroencephalography (EEG) offers a direct and temporally precise method to investigate functional brain connectivity underlying these network properties. In particular, directed connectivity measures derived from multivariate autoregressive modeling, such as the Directed Transfer Function (DTF), allow estimation of directional information flow between neural signals. Prior EEG studies in ADHD have reported altered synchronization patterns and disrupted functional connectivity, particularly in resting‐state conditions, which are thought to reflect intrinsic network instability and maturational delay (Liu et al. 2015; Alba et al. 2016; Janssen et al. 2017). However, most studies have focused on group‐level differences in network metrics, with relatively limited attention to how these metrics relate to clinically relevant behavioral dimensions, including both executive function and motor performance.

A critical limitation in the literature is the relative isolation of behavioral and neurophysiological domains. While executive dysfunction and NSS have each been linked to neural abnormalities, integrative studies that simultaneously examine their relationship with functional network organization remain scarce. This gap is particularly important given that both domains may reflect different manifestations of a shared disruption in network integration. Establishing direct associations between graph‐theoretical metrics and multidimensional behavioral measures may therefore provide a more mechanistic account of how large‐scale brain organization gives rise to observable clinical variability.

An additional dimension that warrants systematic investigation is biological sex. ADHD exhibits well‐documented sex differences in prevalence, symptom profile, and developmental trajectory. Neurophysiological evidence suggests that males and females may exhibit distinct patterns of brain organization and connectivity, even in typical development (Clarke et al. 2001, 2003). Furthermore, developmental EEG studies indicate that age‐ and sex‐dependent changes in brain dynamics may contribute to heterogeneity in ADHD (Poil et al. 2014). Despite this, sex‐specific analyses of brain–behavior relationships, particularly within a network neuroscience framework, remain limited.

Growing EEG‐based connectivity studies suggest that ADHD is characterized not only by altered functional interactions among distributed cortical regions but also by impaired adaptive reconfiguration of large‐scale brain networks. Using the phase‐locking value (PLV) to quantify phase synchronization, Michelini et al. (2019) reported atypical task‐related functional connectivity during cognitive control not only in adolescents and adults with persistent ADHD but also in individuals whose clinical symptoms had remitted. Consistent with this interpretation, Chen et al. (2022) demonstrated that young children with ADHD exhibited atypical resting‐state functional connectivity together with attenuated task‐related modulation of functional networks during inhibitory control. Specifically, compared with typically developing children, the ADHD group showed reduced flexibility in dynamically increasing phase synchronization during cognitive demands, indicating an impaired capacity to efficiently recruit and reorganize distributed neural assemblies in response to changing task requirements. The persistence of these aberrant connectivity patterns despite symptomatic improvement suggests that altered functional network organization may represent a stable neurophysiological endophenotype rather than merely a consequence of current symptom expression. Collectively, these findings support the notion that ADHD involves disrupted large‐scale network integration and reduced adaptive flexibility of functional communication, whereby inefficient synchronization and deficient dynamic reconfiguration of frontoparietal control networks may contribute to persistent impairments in executive functioning and inhibitory control across development.

Despite differences in imaging modalities and analytical approaches, accumulating evidence converges on the view that ADHD is fundamentally associated with atypical large‐scale brain network organization. In healthy development, resting‐state functional networks undergo progressive maturation, characterized by increased integration, strengthened hub architecture, and more efficient information transfer, reflecting the emergence of an optimized balance between network segregation and integration (Cai et al. 2018). In contrast, resting‐state EEG studies have demonstrated that children with ADHD exhibit disrupted rich‐club organization, indicating weakened connectivity among highly interconnected hub regions that form the backbone of efficient neural communication. Such alterations suggest reduced global integration (GE) and impaired coordination among large‐scale functional networks (Ahmadi et al. 2021). Complementary evidence from resting‐state fMRI further indicates that ADHD is associated with altered whole‐brain network topology, including reduced GE and atypical patterns of network reorganization, consistent with diminished functional plasticity and less efficient information processing across distributed brain systems (Tang et al. 2022). Although EEG, fMRI, and fNIRS capture distinct neurophysiological signals and operate at different temporal scales, these multimodal findings consistently support the notion that ADHD reflects a disorder of large‐scale network maturation rather than isolated regional dysfunction. Mechanistically, impaired development of hub connectivity, reduced network integration, and diminished adaptive reorganization are likely to compromise the efficient exchange of information among executive control, attentional, and default mode networks, thereby contributing to the persistent cognitive and behavioral impairments that characterize ADHD.

ADHD is increasingly recognized as a disorder arising from disrupted interactions among distributed brain networks rather than dysfunction confined to isolated cortical regions. Evidence accumulated from multimodal neuroimaging approaches, including structural MRI, resting‐state functional MRI (rs‐fMRI), and EEG, indicates that alterations in the organization and communication of large‐scale neural systems constitute a core neurobiological feature of the disorder. Structural connectome studies have reported impaired network integration, disrupted hub organization, and altered topological properties, suggesting compromised efficiency of information transfer across the brain. Complementary findings from rs‐fMRI consistently reveal atypical functional interactions involving the default mode, frontoparietal executive, salience, and cortico‐striatal networks, all of which play critical roles in attention regulation, executive control, cognitive flexibility, and behavioral inhibition. Resting‐state EEG investigations further support the concept of network‐level dysfunction by demonstrating abnormal synchronization and information exchange, particularly across frontal, frontocentral, and centroparietal regions. Nevertheless, the direction and spatial distribution of these connectivity alterations remain heterogeneous across studies, reflecting differences in participant characteristics, developmental stage, medication status, recording duration, analytical methodology, and the connectivity metrics employed. These methodological variations have complicated the identification of robust electrophysiological biomarkers capable of reliably characterizing ADHD‐related network dysfunction.

Taken together, these findings underscore an important gap in the current ADHD connectivity literature. While numerous studies have reported altered functional brain networks, relatively little is known about the methodological robustness of these findings or the extent to which connectivity estimates remain stable across different temporal scales, frequency bands, and connectivity estimators. Moreover, most previous investigations have focused primarily on identifying group differences in network topology, whereas considerably less attention has been devoted to linking large‐scale functional organization with both executive and motor functions within a sex‐informed framework. Addressing these limitations requires an integrative approach that combines high‐temporal‐resolution electrophysiological recordings, quantitative network analysis, multidimensional behavioral assessment, and systematic evaluation of methodological reliability.

Accordingly, the present study investigated resting‐state EEG functional connectivity using a clinically practical recording protocol designed for children with ADHD, who often experience difficulty remaining motionless during prolonged EEG acquisition. Rather than relying on long recordings obtained with high‐density laboratory EEG systems, we analyzed 1 min of eyes‐closed resting‐state EEG acquired using a portable 14‐channel Emotiv device. Functional connectivity was estimated using four complementary measures representing distinct aspects of neural communication: the DTF, which quantifies directed information transfer, and the Phase Lag Index (PLI), weighted Phase Lag Index (wPLI), and debiased weighted Phase Lag Index (dwPLI), which estimate phase‐based synchronization while minimizing the influence of volume conduction and zero‐lag interactions. Connectivity networks were computed using 50% overlapping sliding windows of 1‐, 2‐, and 4‐s durations to evaluate the influence of temporal resolution on network estimation. Analyses were performed across multiple frequency ranges, including delta, theta, theta1, theta2, alpha, alpha1, alpha2, alpha3, beta, and broadband activity. Beyond conventional graph‐theoretical metrics, edge‐wise connectivity patterns were also examined to identify network connections that remained consistently discriminative across varying thresholds, window lengths, frequency bands, and connectivity estimators. This comprehensive analytical framework enabled simultaneous evaluation of the neurophysiological characteristics of ADHD‐related functional networks and the methodological robustness of the derived connectivity biomarkers.

The present study therefore had two complementary objectives. First, we examined alterations in resting‐state functional brain network organization in children with ADHD relative to typically developing controls and investigated how graph‐theoretical network properties relate to executive performance (Stroop task), neurological soft signs (Physical and Neurological Examination for Soft Signs [PANESS]), and ADHD symptom severity. Second, we systematically evaluated the reliability of connectivity estimation across different temporal scales, frequency bands, and connectivity approaches in order to identify methodological conditions that yield the most robust and reproducible network measures from short‐duration, low‐density EEG recordings. Particular emphasis was placed on determining whether directed information flow estimated by DTF provides more reliable connectivity estimates than phase‐based measures (PLI, wPLI, and dwPLI), as assessed by the proportion of graph metrics achieving excellent test–retest reliability based on intraclass correlation coefficients (ICC).

Based on previous evidence and the objectives of the present study, the following hypotheses were formulated:

**H1‐ Network topology differences**. Children with ADHD will exhibit altered global brain network organization characterized by reduced GE, LE, CC, and network density (Den), together with increased modularity (Q), reflecting reduced network integration and enhanced functional segregation.
**H2‐ Executive function**. Reduced network integration (lower GE, LE, and CC) will be associated with poorer executive performance, reflected by longer Stroop completion times, higher error rates, and increased self‐correction behavior.
**H3‐ Sensorimotor function**. Greater neurological soft signs (higher PANESS scores) will be associated with reduced network integration, particularly lower LE and Den, reflecting disrupted sensorimotor network organization.
**H4‐ Clinical symptom severity**. Greater ADHD symptom severity will be associated with reduced network integration (GE, LE, and CC) and increased modularity (Q), indicating progressively disrupted large‐scale functional organization.
**H5‐ Sex‐dependent network organization**. Brain–behavior associations will differ between boys and girls, with girls exhibiting more specific and stronger connectivity–behavior relationships, whereas boys will demonstrate more spatially distributed alterations in functional network topology.
**H6‐ Methodological robustness of connectivity estimation**. The reliability of resting‐state EEG connectivity will depend on both temporal resolution and frequency band, with specific combinations of window length and oscillatory band yielding more stable graph‐theoretical metrics than others. Furthermore, we hypothesize that directed connectivity estimated by DTF will demonstrate superior methodological robustness compared with phase‐based connectivity measures (PLI, wPLI, and dwPLI), producing a greater proportion of graph metrics with excellent ICC values across repeated epochs. Such findings would indicate that modeling the temporal directionality of information transfer provides more reproducible estimates of functional brain organization than approaches based solely on inter‐electrode phase relationships.

## 2 Methods

### 2.1 Participants and Clinical Assesments

The study drew from a volunteer pool at the Child and Adolescent Psychiatry Outpatient Clinic of Selçuk University Faculty of Medicine, enrolling 41 children diagnosed with ADHD (including 11 girls) and 42 typically developing controls (including 10 girls), all aged between 6 and 12 years. Diagnostic clarity was achieved through the Turkish adaptation of the Kiddie Schedule for Affective Disorders and Schizophrenia for School‐Age Children, Present and Lifetime Version (K‐SADS‐PL‐DSM‐5‐T). These structured dialogues with both children and parents culminated in a consensus diagnosis by experienced child psychiatrists. Reflecting a commitment to ethical rigor, the study received formal approval from the Selçuk University Medical Faculty Hospital Ethics Committee (Protocol No: 2021/477).

Participation was reserved for children within the 6–12 age bracket who demonstrated cognitive functioning congruent with typical development, as evidenced by clinical history, academic performance, and behavioral observations. To ensure a clear neurophysiological signal, candidates were excluded if they possessed chronic medical conditions, a history of severe head trauma, or had received pharmacological treatment for ADHD or other psychiatric conditions within the preceding six months. While comorbid oppositional defiant or conduct disorders were permitted within the ADHD cohort to reflect clinical reality, any other additional psychiatric or neurological disorders served as grounds for exclusion.

A multidimensional assessment framework was employed to capture the nuances of the pediatric mind. Motor precision and neurological integrity were quantified using the PANESS, while behavioral symptomatology was mapped through the Turgay Disruptive Behavior Rating Scale for Parents (T‐DSM‐IV). In tandem with these behavioral measures, resting‐state EEG recordings were acquired under eyes‐closed conditions to unveil the brain's functional architecture. A rich set of socio‐demographic data, ranging from parental education and employment to family psychiatric history, provided a contextual backdrop, with socioeconomic status qualitatively inferred from parental background.

The **K‐SADS‐PL‐DSM‐5‐T** served as the diagnostic cornerstone; a semi‐structured interview revised for DSM‐5 criteria, its validity and reliability within the Turkish population are well‐established (Ünal et al. 2019). Behavioral profiles were further refined using the **T‐DSM‐IV‐S**, a 41‐item parent‐report scale that assesses attention, hyperactivity, and conduct symptoms with validated precision.

To explore the “soft” signatures of neurological maturation, the **PANESS,** originally conceived by Guy (1976) and refined by Denckla (1985), was administered by two child psychiatrists. This instrument is a sensitive herald of subtle motor deficits (VITIELLO et al. 1989), and while Turkish normative values for timed movements remain elusive, its reliability in this population is documented (Uslu et al. 2007; Yurtbaşı et al. 2018).

Finally, executive prowess was gauged via the **Stroop Test** from the BILNOT‐Child battery, a paradigm standardized for Turkish children (Kılıç et al. 2002; Karakaş et al. 2011). As a gold‐standard measure of cognitive control (MacLeod 1992), the Stroop task captures the interference between automaticity and inhibition. By recording completion times, error rates, and self‐corrections, we obtained a multidimensional portrait of the frontal lobe's capacity for sustained attention and resistance to distraction.

All children in the ADHD group were drug‐naïve; had combined‐type ADHD diagnosed by experienced child and adolescent psychiatrist authors of this study according to Turgay‐DSM‐IV criteria; and had no psychiatric comorbidity identified during the clinical evaluation. Regarding cognitive ability, formal IQ testing was not included in the original study protocol. However, all participants underwent comprehensive clinical assessment before enrollment, and only children considered to have age‐appropriate cognitive functioning by the evaluating child and adolescent psychiatrists were included in the study.

Demographic characteristics and clinical symptom scores are summarized in Table 1. Age and clinical measures were compared using independent‐samples t‐tests. Consistent with the sex‐stratified analytical framework adopted in this study, statistical comparisons were performed separately for HC‐boys versus HC‐girls, ADHD‐boys versus ADHD‐girls, HC‐boys versus ADHD‐boys, and HC‐girls versus ADHD‐girls. Clinical symptom severity was assessed using the Turgay DSM‐IV‐Based Child and Adolescent Disruptive Behavior Disorders Screening and Rating Scale. No significant sex‐related differences were observed in either the inattention or hyperactivity scores within the HC or ADHD groups, indicating comparable ADHD symptom profiles between boys and girls within each diagnostic category. In contrast, both inattention and hyperactivity scores were significantly higher in children with ADHD than in their sex‐matched healthy controls (HCs), confirming the expected clinical differences between the ADHD and HC groups.

**TABLE 1:** Demographic characteristics and clinical symptom profiles of the study participants. Participant characteristics are presented separately for HC‐boys, HC‐girls, ADHD‐boys, and ADHD‐girls.

Group	Age	Inattention	Hyperactivity	ODD	CD
HC‐boys (*n* = 32)	9.47 ± 1.67	4.44 ± 4.47	3.66 ± 2.92	0.13 ± 0.34	0.40 ± 0.70
HC‐girls (*n* = 10)	9.90 ± 1.97	4.50 ± 4.35	3.00 ± 2.00	4.60 ± 3.89	2.74 ± 3.12
ADHD‐boys (*n* = 31)	8.90 ± 1.99	14.03 ± 5.43	14.55 ± 6.61	10.68 ± 6.19	12.53 ± 8.31
ADHD‐girls (*n* = 10)	9.40 ± 2.07	16.80 ± 8.39	10.40 ± 5.82	11.60 ± 9.00	13.00 ± 3.74
*p*‐value (boys vs. girls in HC)	0.46	0.96	0.51	*	*
*p*‐value (boys vs. girls in ADHD)	0.50	0.22	0.10	0.71	0.82
*p*‐value (HC vs. ADHD in boys)	0.22	**	**	**	*
*p*‐value (HC vs. ADHD in girls)	0.58	**	**	*	*

*Note*: Age and clinical symptom scores are reported as mean ± standard deviation (SD), whereas participant numbers were presented as *n*. Clinical symptom scores were obtained using the Turgay DSM‐IV‐Based Child and Adolescent Disruptive Behavior Disorders Screening and Rating Scale. The *p*‐values correspond to independent‐samples *t*‐tests comparing HC‐boys versus ADHD‐boys and HC‐girls versus ADHD‐girls, respectively. CD, conduct disorder; ODD, oppositional defiant disorder. * denotes *p* < .05, ** denotes *p* << .05

### 2.2 EEG Data Acquisition and Preprocessing

**FIGURE 1:**
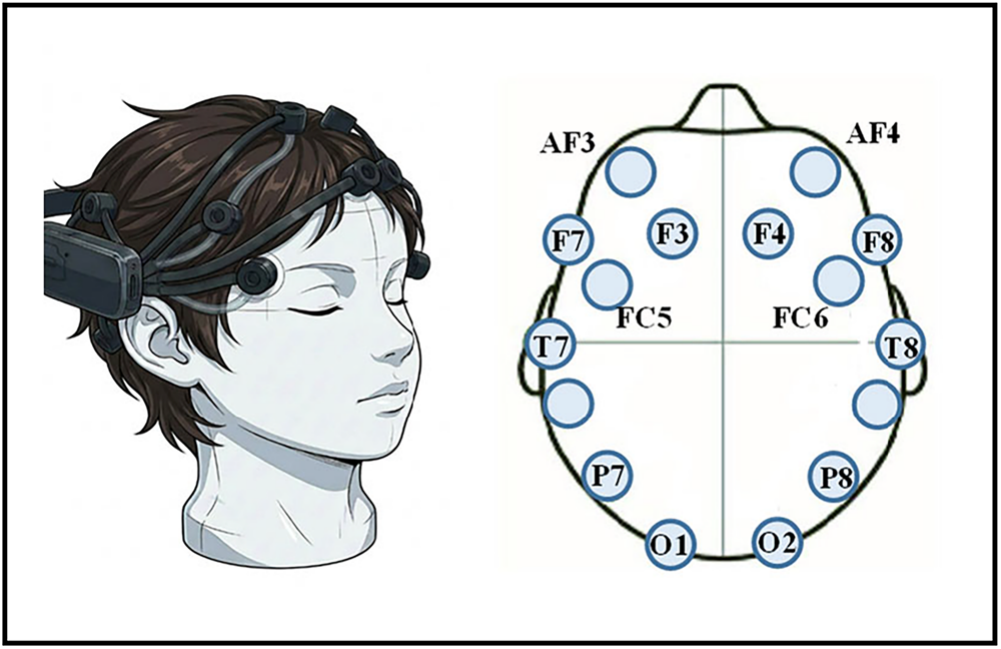
Schematic representation of Emotive headset and electrode placement on scalp surface.

EEG activity was recorded using the Emotiv EPOC headset, a wireless system equipped with 16 gold‐plated electrodes (M1 and M2 were references). Electrode placement adhered to the International 10–20 system, covering the sites AF3, F7, F3, FC5, T7, P7, O1, O2, P8, T8, FC6, F4, F8, and AF4 as shown in Figure 1. The mastoid electrode M1 served as the ground reference, whereas M2 functioned as the feed‐forward reference.

Participants were seated comfortably and instructed to keep their eyes closed during a 1‐min resting‐state recording. Signals were sampled at 256 Hz. Prior to connectivity analyses, continuous raw EEG data recorded via the 14‐channel Emotiv EPOC headset (AF3, F7, F3, FC5, T7, P7, O1, O2, P8, T8, FC6, F4, F8, and AF4) underwent a structured preprocessing pipeline. All EEG preprocessing procedures were performed in MATLAB (MathWorks Inc., Natick, MA, USA) using EEGLAB functions and custom‐developed scripts. To eliminate power‐line contamination and high‐frequency instrumental noise, a second‐order zero‐phase notch filter (49–51 Hz) and a non‐causal band‐pass filter (0.5–45 Hz) were sequentially applied. Following filtering, standardized channel labels were assigned according to the International 10–20 electrode placement system, and 3D spatial coordinates were computed utilizing the standard‐1005 template (standard_1005.elc) to provide accurate spatial topography for subsequent graph‐theoretical computations.

Artifact mitigation was subsequently performed using a custom multi‐channel implementation of Artifact Subspace Reconstruction (ASR). Briefly, a calibration reference covariance matrix was estimated from a clean segment of each recording using overlapping 0.5‐s windows (50% overlap). Principal Component Analysis (PCA) was applied to map the dominant signal subspace, defining component‐specific rejection thresholds at five standard deviations from the reference distribution. During sliding‐window processing, components exceeding these geometric thresholds were flagged as artifact‐contaminated and selectively suppressed, capping the maximum allowable component rejection at 40% of the total rank to systematically preserve physiological neural activity. Cleaned data streams were then reconstructed back into sensor space via inverse projection. No additional channel rejection, interpolation, re‐referencing, or current source density (CSD) transformation was performed.

### 2.3 Graph‐Theoretical EEG Analysis: Functional Connectivity Estimation and Network Construction

To comprehensively evaluate resting‐state functional brain organization in ADHD, functional connectivity was estimated using four complementary approaches representing distinct models of neural communication: the DTF, which quantifies directed information flow, together with the PLI, wPLI, and dwPLI, which estimate phase‐based synchronization while minimizing the influence of volume conduction. The theoretical background and mathematical formulation of each connectivity estimator are presented in the following subsections.

For each participant, 1 min of eyes‐closed EEG was segmented using 50% overlapping sliding windows of 1‐, 2‐, and 4‐s durations. Functional connectivity matrices were independently computed for each segment across ten frequency ranges (delta, theta, theta1, theta2, alpha, alpha1, alpha2, alpha3, beta, and broadband/full‐band activity). This procedure yielded a series of weighted 14 × 14 connectivity matrices for every participant, connectivity estimator, frequency band, temporal window, and EEG segment.

To investigate the influence of network density on graph topology, proportional thresholding was applied independently to each weighted connectivity matrix using sparsity levels of 10%, 15%, 20%, 25%, and 30%. Thresholding retained the strongest functional connections while ensuring comparable network density across participants. Both weighted thresholded adjacency matrices and their corresponding binary representations were generated for subsequent graph‐theoretical analyses.

Three complementary levels of network representation were subsequently evaluated. First, the original weighted connectivity matrices (raw connectivity matrices) were analyzed to examine continuous connectivity strength. Second, proportional thresholding produced weighted adjacency matrices that preserved connection weights while controlling network density. Finally, binary adjacency matrices were generated to characterize network topology independently of connection magnitude. In addition to conventional graph‐theoretical metrics, edge‐level analyses were performed for all three matrix representations, enabling identification of functional connections that consistently differentiated ADHD and typically developing children across different thresholds, frequency bands, temporal scales, and connectivity estimators.

Because resting‐state EEG connectivity estimated from short recordings may vary considerably across temporal segments, methodological reliability was evaluated prior to statistical group comparisons. For each connectivity estimator, graph‐theoretical metric, frequency band, temporal window, and threshold level, ICC was calculated across all sliding‐window segments. Reliability was classified according to established ICC criteria, and only graph metrics demonstrating at least fair reliability were considered for subsequent statistical analyses. Particular emphasis was placed on identifying metrics exhibiting good‐to‐excellent reliability, as these represent the most reproducible candidate biomarkers.

Statistical analyses were performed on individual segment‐level graph metrics without averaging across segments. Linear mixed‐effects models (LMEs) were employed to account for within‐subject repeated measurements while preserving temporal variability. Separate LME models were used to evaluate within‐group and between‐group differences, and all resulting *p*‐values were corrected for multiple comparisons using the Benjamini–Hochberg false discovery rate (FDR) procedure. Graph‐theoretical measures simultaneously satisfying both methodological reliability (good‐to‐excellent ICC) and statistical significance (p_fdr_ < 0.05) were subsequently selected for detailed edge‐level analyses. These analyses identified robust functional connections that remained consistently altered across multiple temporal windows, frequency bands, threshold levels, and connectivity estimation methods.

Graph‐theoretical analysis was performed using the Brain Connectivity Toolbox (BCT; https://sites.google.com/site/bctnet/). The principal network measures included GE clustering, coefficient (CC), LE, modularity (*Q*_mod_), transivitity (*T*_tran_), assortativity (*R*_as_) and rich‐club coefficient (R_cc_). Together, these complementary measures characterize functional segregation, GE, network resilience, hierarchical organization, and modular structure, providing a comprehensive description of large‐scale brain network organization in ADHD (Bullmore and Sporns 2012; Rubinov and Sporns 2010).

#### 2.3.1 Directed Transfer Function

Functional interactions between EEG channels were quantified using the DTF, a frequency‐domain measure derived from the Multivariate Autoregressive Model (MVAR) framework. DTF estimates directional influence between signals based on Granger causality principles, thereby characterizing the flow of information across cortical regions. For each 2‐s EEG epoch, directional connectivity between electrode pairs was calculated over the entire 1‐min recording period. The causal interaction between channels *x* and *y* was computed as follows:

$$\;\mathrm{DT}F_{xy}=\frac{{\left|H\left(f\right)\right|}^{2}}{\sum_{m=1}^{k}{\left|H_{x,m}\left(f\right)\right|}^{2}}$$

where H(f) denotes the transfer function of the MVAR model at frequency f. This formulation allows DTF to capture the spectral distribution of directed interactions across all channels rather than being restricted to a single source node. Connectivity estimation was implemented using the eConnectome toolbox, which incorporates adaptive MVAR modeling (He et al. 2013), with the ARfit package employed for autoregressive coefficient estimation (Neumaier and Schneider 2001; Schneider and Neumaier 2001).

#### 2.3.2 PLI, wPLI, and dwPLI

To complement the directed connectivity analysis obtained using the DTF, three phase‐based functional connectivity measures were employed: the PLI, the wPLI, and the dwPLI. Unlike DTF, which estimates the direction of information flow between EEG channels, these measures quantify the consistency of non‐zero phase relationships between pairs of signals and are specifically designed to reduce the influence of volume conduction and common reference effects. The PLI quantifies the consistency with which one signal leads or lags another by evaluating the sign of the instantaneous phase difference. Considering the phase difference (Δφ(t)) between two EEG signals ($S_{x},\;S_{y}$), the PLI is defined as follows:

$$\mathrm{PLI}=\left|\langle \mathrm{sign}\left(\sin\left(\Delta \varphi \left(t\right)\right)\right)\rangle \right|$$

where ⟨·⟩ denotes temporal averaging. PLI ranges from 0 to 1. with values close to zero indicating either no phase synchronization or synchronization centered around zero phase lag, whereas values approaching one indicate highly consistent non‐zero phase differences. Because zero‐lag synchronization is ignored, PLI substantially reduces spurious connectivity arising from volume conduction. Although PLI effectively suppresses zero‐lag interactions, it treats all phase differences equally regardless of their magnitude. To improve sensitivity, the wPLI assigns greater weight to phase differences associated with larger imaginary components of the cross‐spectrum. It is computed as follows:

$$\mathrm{wPLI}=\frac{\left|\langle \left|\mathrm{im}\left(S_{xy}\right)\right||\mathrm{sign}\left(\mathrm{im}\left(S_{xy}\right)\right)\rangle \right|}{\langle \left|\mathrm{im}\left(S_{xy}\right)\right|\rangle }$$

where $\mathrm{im}(S_{xy})$ denotes the imaginary component of the cross‐spectrum between signals *x* and *y*. By emphasizing phase differences with larger imaginary values, wPLI is less susceptible to noise and provides a more stable estimate of functional connectivity than conventional PLI. Because wPLI may exhibit positive bias when calculated from relatively short data segments, the dwPLI was additionally employed. The debiasing procedure removes the sample‐size‐dependent bias inherent in wPLI while preserving its robustness against volume conduction. Consequently, dwPLI provides more accurate connectivity estimates when the number of observations is limited, making it particularly suitable for analyses based on short EEG epochs.

In the present study, PLI, wPLI, and dwPLI were calculated for all channel pairs within each frequency band and time window. These complementary phase‐based measures were subsequently compared with DTF to evaluate how different connectivity estimators influence graph‐theoretical network properties and their reliability across varying temporal resolutions. Only graph‐theoretical measures that simultaneously demonstrated acceptable methodological reliability (ICC) and statistically significant group effects after FDR correction were retained for subsequent edge‐level analyses.

### 2.4 Statistical Analysis

To assess the reliability of the seven graph‐theoretical connectivity indices (GE, CC, LE, *Q*_mod_, *T*_tran_, *R*_as_, and *R*_cc_), ICCs were computed from connectivity estimates derived from overlapping 1‐, 2‐, and 4‐s EEG epochs (segments), separately for all connectivity estimators (DTF, PLI, wPLI, and dwPLI); nine specified frequency bands (delta, theta, theta1, theta2, alpha, alpha1, alpha2, alpha3, and beta) as well as fullband, and five proportional network thresholds (0.10, 0.15, 0.20, 0.25, and 0.30). Reliability was evaluated separately for each of the six study groups (ADHD‐all, HC‐all, ADHD‐girls, ADHD‐boys, HC‐girls, and HC‐boys), providing an index of measurement precision specific to each diagnosis‐by‐sex subgroup. ICCs were computed in MATLAB using a custom implementation of the classical Shrout and Fleiss (1979) framework, specifically the ICC(3.k) model, which is appropriate for fixed measurement conditions and nonaveraged observations across segments. Reliability values were interpreted using conventional thresholds (< 0.40 poor; 0.40–0.60 fair; .60–0.075 good; ≥ 0.75 excellent) (Hardmeier et al. 2014; Jin et al. 2011; Kuntzelman and Miskovic 2017). ICC served as a gatekeeping criterion: only (metric × epoch length × connectivity method × frequency band × threshold) combinations demonstrating at least good‐to‐excellent reliability in HC‐all group were considered eligible for subsequent inferential analyses. As in prior work, ICC was preferred over simple correlation‐based measures because it explicitly partitions variance into between‐subject, within‐subject, and residual components, thereby capturing both correspondence and absolute agreement between repeated (segment‐level) measurements, a property particularly important given the substantial inter‐epoch variability and potential scaling biases characteristic of short‐epoch EEG connectivity estimates.

Sex‐related comparisons within each diagnostic group (HC‐girls vs. HC‐boys and ADHD‐girls vs. ADHD‐boys) and diagnosis‐related comparisons within sex (ADHD‐girls vs. HC‐girls and ADHD‐boys vs. HC‐boys) were performed using independent‐samples t‐tests. Prior to inferential statistical testing, the reliability of all graph‐theoretical metrics was evaluated using ICCs. Statistical analyses were restricted to graph metrics demonstrating acceptable reliability (ICC ≥ 0.60). To further control for multiple comparisons, all *p*‐values reported in the graph‐theoretical results tables were adjusted using the Benjamini–Hochberg FDR procedure. Therefore, all reported *p*‐values correspond to FDR‐corrected values.

## 3 Results

The findings of this study are unveiled in a structured progression that mirrors our analytical framework. We first evaluated sex‐related differences within each diagnostic group. Because significant sex effects were identified, all subsequent diagnosis‐related comparisons were conducted separately for girls and boys. Building upon this, we evaluated the differences in functional network topology through graph‐theoretical connectivity indices derived from resting‐state EEG. Finally, Spearman rank correlation analyses were employed to map the intricate associations between clinical measures, encompassing NSS, Turgay ratings, and Stroop performance, and the underlying functional architecture of the brain.

To determine whether sex influenced functional connectivity graph metrics within the HC group, between‐sex comparisons (HC‐girls vs. HC‐boys) were performed using LMEs. To ensure that only reliable graph metrics were interpreted, statistical comparisons were restricted to combinations of window length, connectivity method, frequency band, proportional threshold, and graph index that demonstrated acceptable reliability in both subgroups (HC‐girls and HC‐boys). Test–retest reliability of graph‐theoretical metrics was systematically evaluated across all connectivity methods using ICCs. A total of 1050 window–frequency band–threshold–index combinations were examined for each connectivity approach. DTF demonstrated markedly superior reliability, with 557 combinations (53.0%) achieving excellent reliability (ICC ≥ 0.75), 305 (29.0%) showing good reliability, 142 (13.5%) fair reliability, and only 46 (4.4%) classified as poor. In contrast, phase‐based connectivity measures yielded substantially lower reliability. PLI produced only 25 excellent combinations (2.4%), whereas dwPLI and wPLI yielded 19 (1.8%) and 17 (1.6%) excellent combinations, respectively. Moreover, approximately 42% of all combinations derived from the phase‐based methods fell within the poor reliability category, compared with only 4.4% for DTF.

The graph metrics identified as significantly different by the DTF method and phase‐sensitive connectivity approaches, together with the corresponding *p*‐values and effect sizes (Cohen's d), are presented in Tables 3 and 4, respectively. When the same graph metric remained significant across multiple proportional thresholds, the corresponding *p*‐values and effect sizes for each threshold are reported within a single row, separated by commas, to avoid redundancy while preserving all statistically significant findings.

Regarding Table 3, within the HC group, sex‐related differences identified by the DTF method exhibited a clear dependence on both the temporal window length and the frequency band. Overall, the 1‐s window yielded the largest number of significant findings, indicating that short temporal windows were more sensitive to detecting sex‐related differences in graph topology.

At the 1‐s window, significant differences in the delta band were restricted to GE and *Q*_mod_, both observed exclusively at the sparsest network density (threshold = 0.10) with moderate‐to‐large effect sizes (Cohen's *d* ≈ 0.71). Across the theta, theta1, and theta2 bands, significant differences were consistently observed for *Q*_mod_ at thresholds of 0.20 and 0.25, suggesting sex‐dependent alterations in network modular organization within the theta frequency range. In the alpha, alpha1, and alpha3 bands, GE consistently differed between girls and boys at the 0.10 threshold, whereas LE exhibited significant differences only in the alpha2 and alpha3 bands at higher network densities (0.25–0.30), with effect sizes ranging from 0.74 to 0.83. Similarly, transitivity showed significant differences across the alpha2, alpha3, and beta bands at the 0.20 threshold, with the largest effect observed in the alpha2 band (*d* = 0.87). The *R*_cc_ differed significantly only within the alpha2 and alpha3 bands at thresholds of 0.25 and 0.3. In the full‐band analysis, significant differences were again limited to GE and *Q*_mod_, both detected exclusively at the sparsest threshold (0.10). Among all significant findings obtained with the 1‐s window, the largest effect size was observed for *Q*_mod_ in the theta2 band (*d* = 0.90). For the 2‐s window, the overall number of significant findings decreased while remaining predominantly concentrated within the alpha frequency range. LE showed significant differences in both alpha2 and alpha3 bands at thresholds of 0.25 and 0.3, with effect sizes ranging from 0.76 to 0.83. The *R*_cc_ exhibited a consistent pattern across the alpha, alpha1, and alpha2 bands at thresholds of 0.20 and 0.25, with the strongest effect again observed in the alpha2 band. Likewise, *T*_tran_ remained significantly different in the alpha2, alpha3, and beta bands at thresholds of 0.20 and 0.25, reaching its largest effect size within the beta band at the 0.20 threshold. In contrast, CC demonstrated a significant difference only in the alpha2 band at the 0.20 threshold, indicating a relatively limited sensitivity compared with the other graph metrics. At the 4‐s window, significant sex‐related differences became largely confined to alpha‐band network organization. GE consistently differed between girls and boys across the alpha, alpha1, alpha2, and alpha3 bands at the 0.20 threshold, with effect sizes ranging from 0.79 to 0.84, whereas in the beta band significant differences were observed only at the sparser thresholds of 0.10 and 0.15 with comparable effect sizes. Both LE and the *R*_cc_ showed significant differences exclusively in the alpha2 and alpha3 bands at the 0.30 threshold. The largest effect size identified for the 4‐s window was observed for LE in the alpha2 band. Taken together, these findings indicate that sex‐related differences in healthy children were predominantly reflected in network integration (GE), network segregation (LE), and modular organization (*Q*_mod_), with the most consistent effects emerging within the alpha sub‐bands. Moreover, the greatest sensitivity to sex‐related topological differences was achieved using the 1‐s temporal window, whereas longer windows progressively restricted significant findings to alpha‐band network organization.

Regarding Table 3, compared with the DTF‐based analysis results summarized in Table 2 , phase‐sensitive connectivity methods (PLI, wPLI, and dwPLI) exhibited a distinct pattern of sex‐related differences in graph topology. Among these methods, PLI yielded the largest number of significant findings, indicating greater sensitivity to sex‐related topological differences than wPLI and dwPLI under the applied reliability criterion. Although the distribution of significant results varied across connectivity measures, all three phase‐based methods identified significant differences within the full‐band analysis. However, the graph metrics contributing to these differences differed substantially across methods. Specifically, PLI identified significant differences in *Q*_mod_ at the 4‐s window and 0.25 threshold, whereas wPLI detected significant differences only in LE for the 1‐s window at the 0.25 threshold. In contrast, dwPLI revealed significant differences in both LE and CC under the same experimental condition (1‐s window, full‐band, threshold = 0.25). Across all three phase‐based connectivity methods, the largest effect size was observed with the PLI approach, where GE within the alpha band differed between HC‐girls and HC‐boys at the 4‐s window and 0.30 threshold (*d* = 0.99). Notably, unlike the DTF analysis, in which *R*_as_ did not show any significant sex‐related differences, the PLI approach identified significant differences for this metric in both the alpha3 band (1‐s window, threshold of 0.25) and the full‐band analysis (2‐s window, threshold of 0.30), with the latter yielding the larger effect size (*d* = 0.91). Furthermore, while LE and CC did not emerge as common findings across all connectivity measures, both metrics were simultaneously identified only by the dwPLI approach in the full‐band analysis using the 1‐s window and 0.25 threshold, suggesting that this estimator may be particularly sensitive to subtle sex‐related differences in local network organization under short temporal windows. Across all three phase‐based connectivity methods, the largest effect size was observed with the PLI approach, where GE within the alpha band differed between HC‐girls and HC‐boys at the 4‐s window and 0.30 threshold (d = 0.99). Notably, unlike the DTF analysis, in which *R*_as_ did not show any significant sex‐related differences, the PLI approach identified significant differences for this metric in both the alpha3 band (1‐s window, threshold = 0.25) and the full‐band analysis (2‐s window, threshold = 0.30), with the latter yielding the larger effect size (d = 0.91). Furthermore, while LE and CC did not emerge as common findings across all connectivity measures, both metrics were simultaneously identified only by the dwPLI approach in the full‐band analysis using the 1‐s window and 0.25 threshold. Importantly, both metrics exhibited negative effect sizes, indicating higher LE and CC values in HC‐boys than in HC‐girls under these conditions. This finding suggests that the dwPLI estimator may be particularly sensitive to subtle sex‐related differences in local network organization captured within short temporal windows.

**TABLE 2:** Significant sex‐related differences in HC in terms of reliable graph‐theoretical metrics by DTF (ICC ≥ 0.60).

HC‐girls (*reference*) vs. HC‐boys with respect to DTF					
w	Band	Index	Threshold	*p*‐value	Effect size
1	Delta	GE	0.10	0.04	0.71
	Delta	*Q*_mod_	0.10	0.04	0.71
	Theta	*Q*_mod_	0.25	0.03	0.77
	Theta1	*Q*_mod_	0.25	0.03	0.77
	Theta2	*Q*_mod_	0.20	0.01	0.90
	Alpha	GE	0.10, 0.25, 0.30	0.04, 0.03, 0.03	0.70, 0.77, 0.74
	Alpha	*Q*_mod_	0.10, 0.15, 0.25, 0.30	0.03, 0.05, 0.04, 0.03	0.76, 0.69, 0.73, 0.79
	Alpha1	GE	0.10, 0.25, 0.30	0.04, 0.03, 0.03	0.70, 0.77, 0.74
	Alpha1	*Q*_mod_	0.10, 0.15, 0.25, 0.30	0.03, 0.05, 0.04, 0.03	0.76, 0.69, 0.73, 0.79
	Alpha2	LE	0.25, 0.30	0.03, 0.02	0.76, 0.83
	Alpha2	*T*_tran_	0.25, 0.30	0.01, 0.01	0.86, 0.87
	Alpha2	*R*_cc_	0.20, 0.25, 0.30	0.04, 0.03, 0.05	0.73, 0.77, 0.69
	Alpha3	GE	0.10	0.04	0.71
	Alpha3	LE	0.25, 0.30	0.03, 0.04	0.77, 0.74
	Alpha3	*T*_tran_	0.20, 0.25, 0.30	0.04, 0.02, 0.02	0.70, 0.83, 0.85
	Alpha3	*R*_cc_	0.25, 0.30	0.02, 0.03	0.79, 0.77
	Beta	*T*_tran_	0.20	0.03	0.75
	Fullband	GE	0.10	0.04	0.71
	Fullband	*Q*_mod_	0.10	0.04	0.71
2	Alpha	GE	0.10, 0.20, 0.25	0.03, 0.02, 0.05	0.77, 0.79, 0.69
	Alpha	*R*_cc_	0.20, 0.25	0.04, 0.03	0.71, 0.76
	Alpha1	GE	0.10, 0.20, 0.25	0.03, 0.02, 0.05	0.77, 0.79, 0.69
	Alpha1	*R*_cc_	0.20, 0.25	0.04, 0.03	0.71, 0.76
	Alpha2	GE	0.15, 0.30	0.03, 0.04	0.78, 0.73
	Alpha2	LE	0.15, 0.20, 0.25, 0.30	0.04, 0.03, 0.03, 0.02	0.71, 0.78, 0.78, 0.83
	Alpha2	CC	0.20	0.05	0.68
	Alpha2	*T*_tran_	0.10, 0.15, 0.20, 0.25	0.04, 0.04, 0.03, 0.03	0.70, 0.71, 0.78, 0.77
	Alpha2	*R*_cc_	0.20, 0.25, 0.30	0.01, 0.01, 0.01	0.95, 0.96, 0.93
	Alpha3	LE	0.25, 0.30	0.03, 0.04	0.76, 0.73
	Alpha3	*T*_ran_	0.15, 0.20, 0.25	0.01, 0.04, 0.03	0.87, 0.72, 0.76
	Beta	*T*_tran_	0.20, 0.25	0.01, 0.03	0.89, 0.77
4	Alpha	GE	0.20, 0.25	0.03, 0.02	0.76, 0.79
	Alpha1	GE	0.20, 0.25	0.03, 0.02	0.76, 0.79
	Alpha2	GE	0.10, 0.15, 0.20, 0.25, 0.30	0.02, 0.03, 0.02, 0.02, 0.05	0.85, 0.76, 0.84, 0.80, 0.69
	Alpha2	LE	0.15, 0.20, 0.25, 0.30	0.04, 0.0001, 0.0001, 0.01	0.70, 1.03, 1.01, 0.94
	Alpha2	CC	0.20, 0.25, 0.30	0.01, 0.01, 0.02	0.88, 0.88, 0.83
	Alpha2	*R*_cc_	0.15, 0.20, 0.25, 0.30	0.05, 0.01, 0.02, 0.01	0.70, 0.96, 0.83, 0.90
	Alpha3	GE	0.10, 0.15, 0.20	0.04, 0.04, 0.04	0.72, 0.72, 0.71
	Alpha3	LE	0.30	0.04	0.70
	Alpha3	*T*_tran_	0.15, 0.20	0.04, 0.05	0.73, 0.69
	Alpha3	*R*_cc_	0.30	0.02	0.79
	Beta	GE	0.10, 0.15	0.03, 0.05	0.75, 0.68

*Note*: Statistical significance was assessed using LMEs. For graph metrics that remained significant across multiple proportional thresholds, the corresponding *p*‐values and effect sizes (Cohen's d) are reported within a single row as comma‐separated values.

**TABLE 3:** Significant sex‐related differences in HC in terms of reliable graph‐theoretical metrics (ICC ≥ 0.60) by phase‐sensitive connectivity estimators (PLI, wPLI, and dwPLI). Statistical significance was assessed using LMEs.

HC‐girls (*reference*) vs. HC‐boys with respect to PLI, wPLI, dwPLI						
Method	Window	Band	Index	Threshold	*p*‐value	Effect size
PLI	1	Alpha3	*R*_as_	0.25	0.04	0.72
	2	Alpha	GE	0.2	0.05	0.69
		Fullband	*R*_as_	0.3	0.01	0.91
	4	Alpha	GE	0.25, 0.3	0.02, 0.01	0.85, 0.99
		Alpha1	*Q*_mod_	0.3	0.05	0.70
		Fullband	*Q*_mod_	0.25	0.05	0.70
wPLI	1	Alpha	*Q*_mod_	0.1, 0.3	0.04, 0.04	0.72, 0.71
		Fullband	LE	0.25	0.03	−0.74
dwPLI	1	Fullband	LE	0.25, 0.3	0.03, 0.05	−0.77, −0.69
		Fullband	CC	0.25	0.05	−0.70

*Note*: For graph metrics that remained significant across multiple proportional thresholds, the corresponding *p*‐values and effect sizes (Cohen's d) are reported within a single row as comma‐separated values.

Sex‐related differences within the ADHD group are summarized in Table 4. Compared with the HC group, substantially fewer graph‐theoretical metrics exhibited significant sex‐related differences when the DTF connectivity estimator was used, indicating a markedly reduced sensitivity of directed functional connectivity measures to sex effects in ADHD. Within the DTF analysis, significant differences were confined to the 4‐s temporal window, where LE and CC differed between ADHD‐girls and ADHD‐boys in the alpha and alpha1 bands. Notably, all significant DTF findings were consistently observed at the 0.15 proportional threshold, suggesting a stable network‐density dependence that contrasted with the broader threshold distribution observed in the HC group. Furthermore, unlike the HC comparisons, the effect sizes for all significant LE and CC differences were negative, indicating higher values in ADHD‐boys than in ADHD‐girls. In contrast, *Q*_mod_ consistently exhibited positive effect sizes across all significant frequency bands, reflecting the opposite direction of sex‐related differences for this metric. The phase‐based connectivity estimators showed a markedly different pattern. Similar to the HC analysis, dwPLI produced findings that diverged from the other connectivity measures. Specifically, GE demonstrated a negative effect size within the alpha3 band at the 2‐s window, whereas the same metric exhibited a positive effect size in the beta band under identical temporal conditions, suggesting a frequency‐dependent reversal in the direction of sex‐related differences. Although DTF findings were consistently restricted to the 0.15 threshold, the phase‐sensitive estimators (PLI, wPLI, and dwPLI) identified common alterations at different network densities. In particular, LE and CC showed significant differences in the delta band at the 0.20 threshold, with consistently negative effect sizes, indicating higher values in ADHD‐boys than in ADHD‐girls. Conversely, GE exhibited positive effect sizes in the beta band at the 2‐s window and 0.30 threshold, a pattern reproduced by both PLI and dwPLI. Likewise, *Q*_mod_ emerged exclusively in the wPLI and dwPLI analyses, where significant differences were consistently detected within the theta2 band at the 1‐s window (threshold = 0.20) and 2‐s window (threshold = 0.30). Across all connectivity approaches, the largest effect sizes were observed for LE and CC derived from the phase‐sensitive estimators within the delta band, reaching Cohen's *d* values between 0.80 and 0.84. Overall, compared with the HC group, sex‐related differences in ADHD were less numerous, more method‐dependent, and characterized by a greater prevalence of negative effect sizes, particularly for metrics reflecting local network organization. These findings suggest that ADHD alters not only the magnitude but also the direction and spatial organization of sex‐related differences in functional brain network topology.

**TABLE 4:** Significant sex‐related differences in ADHD in terms of reliable graph‐theoretical metrics (ICC ≥ 0.60) by both DTF and phase‐sensitive connectivity estimators (PLI, wPLI, and dwPLI). Statistical significance was assessed using LMEs.

ADHD‐girls (*reference*) vs. ADHD‐boys with respect to connectivity methods						
Method	w	Band	Index	Threshold	*p*‐value	Effect size
DTF	4	Alpha	LE	0.15	0.05	−0.70
		Alpha	CC	0.15	0.04	−0.74
		Alpha1	LE	0.15	0.05	−0.70
		Alpha1	CC	0.15	0.04	−0.74
PLI	1	Delta	LE	0.20	0.02	−0.80
			CC	0.20	0.02	−0.84
		Theta	LE	0.15	0.03	−0.75
		Theta2	GE	0.10	0.04	−0.70
	2	Beta	GE	0.30	0.04	0.72
wPLI	1	Delta	CC	0.15	0.04	−0.70
			CC	0.20	0.02	−0.81
			LE	0.20	0.02	−0.81
		Theta	GE	0.10	0.05	−0.68
			*R*_as_	0.20	0.04	0.71
		Theta2	*Q*_mod_	0.20	0.05	0.68
	2	Theta	*Q*_mod_	0.30	0.05	0.69
		Theta2	*Q*_mod_	0.30	0.03	0.75
		Alpha1	*Q*_mod_	0.10	0.05	0.68
		Alpha3	GE	0.10	0.05	−0.68
dwPLI	1	Delta	LE	0.20	0.02	−0.82
			CC	0.20	0.02	−0.81
		Theta	GE	0.10	0.04	−0.70
			*R*_as_	0.20	0.04	0.70
		Theta2	*Q*_mod_	0.20	0.04	0.71
	2	Theta	*Q*_mod_	0.30	0.04	0.71
		Theta2	*Q*_mod_	0.30	0.03	0.75
		Alpha3	GE	0.10	0.04	−0.70
		Beta	GE	0.30	0.05	0.69
		Fullband	*Q*_mod_	0.25	0.05	0.68

*Note*: For graph metrics that remained significant across multiple proportional thresholds, the corresponding *p*‐values and effect sizes (Cohen's d) are reported within a single row as comma‐separated values.

Having established that sex significantly influenced several graph‐theoretical connectivity metrics within both the HC and ADHD groups, a pooled comparison between all children with ADHD and all HC was not considered appropriate, as such an analysis would potentially obscure sex‐specific network alterations. Instead, subsequent between‐group analyses were performed separately for girls (ADHD girls vs. HC girls) and boys (ADHD boys vs. HC boys). As summarized in Table 5, the DTF approach revealed a remarkably consistent pattern of connectivity alterations between ADHD‐girls and HC‐girls. Across the 1‐s, 2‐s, and 4‐s temporal windows, LE, CC, *T*_tran_, and the *R*_cc_ consistently differed within the alpha2 band at the 0.30 proportional threshold, all exhibiting large negative effect sizes ranging from −1.01 to −1.17, indicating substantially lower values in ADHD‐girls than in healthy girls. The 1‐s window yielded the largest number of significant findings. Within this temporal window, LE demonstrated significant differences across the delta, alpha2, alpha3, beta, and full‐band analyses, consistently at the 0.25 and 0.30 thresholds. Likewise, *R*_cc_ differed significantly within the alpha, alpha1, and alpha2 bands, again at the 0.25 and 0.30 thresholds. Overall, the between‐group comparisons produced considerably larger effect sizes than those observed in the within‐group sex comparisons, and these effects were predominantly negative, indicating a widespread reduction in network segregation and hub organization in ADHD girls relative to healthy girls.

**TABLE 5:** Significant differences between ADHD‐girls and HC‐girls in terms of reliable graph‐theoretical metrics (ICC ≥ 0.60) by DTF and phase‐sensitive connectivity estimators (wPLI and dwPLI).

ADHD‐girls (*reference*) vs. HC‐girls with respect to DTF						
w	Index		Band	Threshold	*p*‐value	Effect size
1	Delta		LE	0.25, 0.30	0.011, 0.023	−1.23, −1.08
	Alpha		*R*_cc_	0.25, 0.30	0.022, 0.042	−1.09, −0.95
	Alpha1		*R*_cc_	0.25, 0.30	0.022, 0.042	−1.09, −0.95
	Alpha2		LE	0.20, 0.25, 0.30	0.02. 0.017, 0.032	−1.11, −1.14, −1.01
			CC	0.20, 0.25, 0.30	0.022, 0.008, 0.013	−1.09, −1.29, −1.20
			*T*_tran_	0.25, 0.30	0.014, 0.011	−1.19, −1.23
			*R*_cc_	0.25, 0.30	0.022, 0.029	−1.09, −1.03
	Alpha3		LE	0.20, 0.25, 0.30	0.016, 0.022, 0.047	−1.16, −1.09, −0.93
			CC	0.25, 0.30	0.01. 0.005	−1.26, −1.37
			*T*_tran_	0.20, 0.25, 0.30	0.021, 0.009, 0.005	−1.1. −1.26, −1.40
	Beta		LE	0.25, 0.30	0.014, 0.044	−1.19, −0.94
			CC	0.20, 0.25, 0.30	0.015, 0.016, 0.007	−1.17, −1.16, −1.33
			*T*_tran_	0.20, 0.25	0.037, 0.010	−0.98, −1.24
	Fullband		LE	0.25, 0.30	0.011, 0.023	−1.23, −1.08
			CC	0.30	0.009	−1.26
2	Alpha2		LE	0.20, 0.30	0.015, 0.033	−1.17, −1.01
			CC	0.20, 0.25, 0.30	0.019, 0.03. 0.016	−1.12, −1.02, −1.16
			*T*_tran_	0.30	0.048	−0.92
			*R*_cc_	0.30	0.024	−1.07
4	Theta		LE	0.20	0.013	−1.19
	Alpha2		LE	0.25, 0.30	0.016, 0.038	−1.15, −0.98
			CC	0.25, 0.30	0.018, 0.017	−1.13, −1.14
			*T*_tran_	0.30	0.036	−0.99
			*R*_cc_	0.30	0.031	−1.02
	Beta		GE	0.10	0.022	−1.09
			LE	0.30	0.030	−1.03
			*T*_tran_	0.30	0.022	−1.09
ADHD‐girls (*reference*) vs. HC‐girls with respect to wPLI						
2		Theta	*Q*_mod_	0.3	0.039	0.97
		Theta2	*Q*_mod_	0.3	0.036	0.99
ADHD‐girls (*reference*) vs. HC‐girls with respect to dwPLI						
2		Theta	*Q*_mod_	0.3	0.035	1.00
		Theta2	*Q*_mod_	0.3	0.041	0.96

*Note*: Statistical significance was assessed using LMEs. When a graph metric remained significant across multiple proportional thresholds, the corresponding *p*‐values and effect sizes (Cohen's d) were presented within a single row as comma‐separated values.

The phase‐sensitive connectivity estimators yielded a much more restricted pattern of significant findings as summarized in Table 5. Notably, wPLI and dwPLI converged on a single common result: *Q*_mod_ differed significantly between ADHD‐girls and HC‐girls only at the 2‐s temporal window, within the theta and theta2 frequency bands, and exclusively at the 0.30 proportional threshold. In contrast to the predominantly negative effect sizes observed with the DTF approach, these differences were characterized by positive effect sizes, suggesting higher modular organization in ADHD‐girls relative to healthy girls when connectivity was estimated using phase‐sensitive measures.

Regarding Table 6, a highly distinctive topological pattern emerged when analyzing functional network reconfigurations between healthy and clinical male cohorts (ADHD‐Boys vs. HC‐Boys). When using DTF, significant between‐group differences were strictly localized within the higher‐frequency (alpha3, beta) networks under the sparsest density as 0.10) configurations in terms of *R*_cc_. In sharp contrast, the phase‐sensitive estimators converged on a complementary topological phenomenon, primarily capturing macro‐level structural adjustments at denser network profiles (e.g., 0.20, 0.25), providing an alteration in modularity index (*Q*_mod_) with negative effect size across the more specific frequency bands such as theta, theta2, alpha, and alpha2.

**TABLE 6:** Significant differences between ADHD‐boys and HC‐boys in terms of reliable graph‐theoretical metrics (ICC ≥ 0.60) by DTF and phase‐sensitive connectivity estimators (PLI, wPLI, and dwPLI).

ADHD‐boys (*reference*) vs. HC‐boy with respect to DTF					
w	Index	Band	Threshold	*p*‐value	Effect size
1	Alpha3	*R*_cc_	0.10	0.04	−0.56
	Beta	*R*_cc_	0.10	0.02	−0.63
ADHD‐boys (*reference*) vs. HC‐boy with respect to PLI					
2	Alpha	*Q*_mod_	0.10	0.05	−0.52
	Alpha2	*Q*_mod_	0.25	0.04	−0.54
ADHD‐boys (*reference*) vs. HC‐boy with respect to wPLI					
1	Fullband	LE	0.25, 0.30	0.05	0.51
2	Theta	*Q*_mod_	0.20, 0.25	0.02	−0.63
	Theta2	*Q*_mod_	0.20	0.03	−0.58
ADHD‐boys (*reference*) vs. HC‐boy with respect to dwPLI					
2	Theta	*Q*_mod_	0.20, 0.25	0.02	−0.63
	Theta2	*Q*_mod_	0.20	0.03	−0.58

*Note*: Statistical significance was assessed using LMEs. When a graph metric remained significant across multiple proportional thresholds, the corresponding *p*‐values and effect sizes (Cohen's d) were presented within a single row as comma‐separated values.

In synthesis, our multi‐threshold exploration suggested distinct topological patterns across cohorts. In typically developing children, sex differences in GE and Q_mod_ were concentrated at the sparsest network density (0.10) within the lower alpha range, whereas differences in LE, T_tran_, and R_cc_ were instead concentrated at denser thresholds (0.20–0.30) within higher alpha bands, suggesting that, unlike integration and modularity, segregation‐ and hub‐related metrics appeared to distinguish the sexes mainly under denser network conditions. Clinical sex differences (ADHD‐boys vs. ADHD‐girls) appeared comparatively attenuated and were concentrated at a moderately sparse threshold (0.15), with ADHD‐boys showing higher segregation (LE, CC) than ADHD‐girls in the alpha and alpha1 bands. Disease‐related reconfigurations also appeared to differ between sexes in their density dependence: The female clinical contrast diverges from this localized right‐hemispheric configuration, most consistently at dense thresholds (0.25–0.30) within the alpha2 band, with additional reductions in LE observed across the delta, alpha3, beta, and fullband networks. The male clinical contrast (HC‐boys vs. ADHD‐boys), by comparison, appeared restricted to a reduction in R_cc_ at the sparsest threshold (0.10) within the alpha3 and beta bands.

To further investigate the potential clinical significance of the identified network alterations, Spearman rank correlation analyses were planned between DTF‐based graph‐theoretical indices (utilizing a 4‐s temporal window) and behavioral measures, including NSS and Stroop Battery scores listed in Table 1. Rather than examining every statistically significant finding independently, candidate connectivity markers were selected according to predefined criteria derived from the overall statistical results. Specifically, priority was given to connectivity combinations (window length, connectivity estimator, frequency band, proportional threshold, and graph metric) that consistently demonstrated significant between‐group differences across multiple analyses, exhibited moderate‐to‐large effect sizes, and satisfied the predefined reliability criterion (ICC ≥ 0.60 in both compared subgroups). This strategy reduced the number of statistical comparisons while emphasizing the most robust and reproducible network features. The results for HC and ADHD patients, stratified by gender, are selectively presented in Tables 7 and 8, respectively.

**TABLE 7:** Spearman rank correlation coefficients (***ρ***) between behavioral/clinical scores and reliable functional connectivity graph metrics in HC‐boys (*n* = 32) and HC‐girls (*n* = 10).

Behavioral/clinical score	Index	Band	Thr	*ρ*	*p*	ICC
**Statistical correlations between clinical scores and connectivity measures with DTF in HC‐boys**						
Block3 errors	CC	Theta2, alpha, alpha1	0.15, 0.20	0.57	0.005	E
	LE	Theta2, alpha, alpha1	0.15, 0.20	0.57	0.005	E
	*Q*_mod_	Theta, theta1, alpha3	0.15	−0.46	0.029	E
	*R*_cc_	Alpha, alpha1	0.15	0.53	0.011	E
Block3‐5 self corrections	GE	Alpha, alpha1	0.20	0.42	0.050	E
	CC	Theta2, alpha, alpha1	0.15, 0.20	−0.57	0.005	E
	LE	Theta2, alpha, alpha1	0.15	−0.42	0.050	E
	*T*_tran_	Alpha2	0.20	−0.42	0.050	E
	*R*_as_	Theta2, fullband	0.15, 0.20	0.50	0.017	E
	*R*_cc_	Alpha2, beta	0.15, 0.20	−0.57	0.006	E
Stroop Block3‐5 completiontime	CC	Alpha2	0.15	−0.48	0.024	E
	LE	Alpha2	0.15	−0.48	0.024	E
	*Q*_mod_	Alpha3	0.15	0.49	0.020	G
	*R*_as_	Alpha3, beta	0.15, 0.20	0.59	0.008	E
Total dysrhythmia	GE	Beta	0.15, 0.20	0.43	0.043	E
	*R*_as_	Beta	0.20	0.45	0.036	E
Total gait and station	GE	Delta, fullband	0.20	−0.51	0.015	E
Total time	GE	Theta, theta1, theta2	0.15, 0.20	0.48	0.024	E
	*Q*_mod_	Theta2, alpha3	0.15, 0.20	0.65	0.001	E
	*R*_as_	Alpha3, beta	0.15, 0.20	0.68	0.001	E
Turgay conduct disorder	CC	Delta, fullband	0.15, 0.20	0.66	0.001	E
	LE	Delta, fullband	0.15	0.69	0.000	G
	*Q*_mod_	Theta2	0.15	−0.43	0.046	E
	*R*_as_	Alpha3, beta	0.15, 0.20	−0.61	0.002	E
Turgay hyperactivity	CC	Delta, fullband	0.15, 0.20	0.52	0.014	E
	LE	Delta, fullband	0.15	0.54	0.010	G
	*R*_as_	Alpha3, beta	0.15	−0.48	0.025	E
Turgay ınattention	CC	Delta, fullband	0.15, 0.20	0.64	0.001	E
	LE	Delta, fullband	0.15	0.65	0.001	G
	*R*_as_	Alpha3, beta	0.15, 0.20	−0.67	0.001	E
	*Q*_mod_	Theta2, alpha, alpha1	0.15, 0.20	−0.61	0.003	E
Turgay oppositional defiant disorder	CC	Alpha3	0.2	0.42	0.049	E
	*Q*_mod_	Beta	0.2	−0.42	0.049	G
**Statistical correlations between clinical scores and connectivity measures with DTF in HC‐girls**						
Block5 errors	*R*_as_	Alpha2	0.20	1	0.03	E
Block3‐5 self corrections	GE	Delta, fullband	0.20	−1	0.03	G
	CC	Alpha2	0.15	1	0.04	G
	LE	Alpha2	0.15	1	0.02	G
	*Q*_mod_	Alpha2	0.15	−1	0.01	G
	*R*_as_	Alpha2	0.20	1	0.03	E
	*R*_cc_	Beta	0.20	1	0.03	E
Stroop Block2‐5 completion Time	GE	Theta2, alpha2	0.15, 0.20	−1	0.03	E
	GE	Delta, fullband	0.20	1	0.03	G
	CC	Alpha2	0.15	−1	0.04	G
	LE	Alpha2	0.15	−1	0.02	G
	*Q*_mod_	Alpha2	0.15	1	0.01	G
	*R*_as_	Alpha2, beta	0.20	1	0.03	E
	*R*_cc_	Beta	0.20	−1	0.03	E
	*T*_tran_	Theta2	0.20	1	0.03	G
Total dysrhythmia	CC	Alpha2	0.15	0.96	0.04	G
	*R*_as_	Beta	0.20	−0.96	0.04	E
Total gait and station	GE	Theta, theta1	0.15	−0.98	0.02	E
	CC	Alpha2	0.15	−0.97	0.03	G
	LE	Alpha2	0.15	−0.97	0.03	G
Total time	GE	Theta, theta1	0.15, 0.20	−0.99	0.01	E
Turgay conduct disorder	GE	Theta2, alpha2	0.15, 0.20	1	0.03	E
Turgay hyperactivity	*R*_as_	Alpha2	0.20	−1	0.03	E
Turgay oppositional defiant disorder	GE	Theta, theta1, alpha, alpha1, alpha2	0.15, 0.20	1	0.03	E

*Note*: Results were selectively presented for the 4‐s temporal window utilizing DTF. Multiple parameter combinations were combined in the same row when they were equivalent at the same index and multiple thresholds and provided the same directional correlation coefficient. G refers to a good category in ICC values satisfying 0.60 ≤ ICC < 0.75, and E refers to an excellent category in ICC values satisfying ≥ 0.75. ***Thr*** refers to the proportional threshold.

**TABLE 8:** Spearman rank correlation coefficients (***ρ***) between behavioral/clinical scores and reliable functional connectivity graph metrics in ADHD‐boys (*n* = 31) and ADHD‐girls (*n* = 10).

Behavioral/clinical score	Index	Band	Thr	*ρ*	*p*	ICC
**Statistical correlations between clinical scores and connectivity measures with DTF** **in ADHD‐boys**						
Block3 errors	CC	Alpha3	0.20	0.39	0.049	G
	LE	Beta	0.20	−0.43	0.028	G
Block3‐5 self corrections	GE	Delta, fullband	0.15	0.44	0.024	G
	*T*_tran_	Alpha2	0.20	0.46	0.018	G
	*R*_as_	Theta2, alpha2	0.15, 0.20	0.63	0.001	E
	*R*_cc_	Alpha3	0.20	0.40	0.044	E
Stroop Block1‐5 completion time	GE	Beta	0.20	0.46	0.019	E
	*Q*_mod_	Beta	0.20	0.42	0.031	G
	*T*_tran_	Alpha2	0.20	0.42	0.036	G
	*R*_as_	Theta2, alpha2	0.20	0.62	0.001	E
Total dysrhythmia	*R*_as_	Alpha3	0.30	0.39	0.047	E
Total gait and station	*R*_cc_	Alpha3, beta	0.20, 0.25, 0.30	0.52	0.006	E
	*T*_tran_	Alpha3	0.25	0.40	0.045	E
Turgay conduct disorder	CC	Delta, fullband	0.20	0.41	0.040	G
Turgay hyperactivity	*T*_tran_	Beta	0.15	0.47	0.016	G
Turgay ınattention	GE	Alpha2	0.15, 0.20	0.61	0.001	E
	*T*_tran_	Alpha2	0.15, 0.20	0.51	0.007	E
	*R*_as_	Alpha2	0.15, 0.20	0.49	0.012	E
Turgay oppositional defiant disorder	CC	Beta	0.15	0.44	0.023	G
	LE	Beta	0.15	0.40	0.041	G
	*T*_tran_	Beta	0.15	0.41	0.038	G
**Statistical correlations between clinical scores and connectivity measures with DTF** **in ADHD‐girls**						
Block3‐5 self corrections	GE	Alpha3, beta	0.20, 0.30	−0.91	0.02	E
	LE	Alpha3	0.30	−0.88	0.04	E
	*R*_as_	Delta, fullband	0.30	−0.99	0.01	E
Stroop Block2‐5 completiontimE	*R*_as_	Alpha, alpha1	0.25	0.89	0.03	E
	*R*_as_	Delta, fullband	0.30	−0.89	0.03	G
Total gait and station	GE	Alpha, alpha1, alpha2	0.15	0.93	0.02	E
	*R*_as_	Alpha2	0.20, 0.25, 0.30	0.93	0.02	E
	*R*_cc_	Alpha3	0.25	0.93	0.02	E
	*Q*_mod_	Beta	0.25	0.87	0.03	E
Total time	*R*_as_	Alpha, alpha1	0.25	0.89	0.03	E
Turgay conduct disorder	LE	Alpha3	0.3	0.90	0.03	E

*Note*: Results are selectively presented for the 4‐s temporal window utilizing DTF. Multiple parameter combinations were combined in the same row when they were equivalent at the same index and multiple thresholds and provided the same directional correlation coefficient. G refers good category in ICC values satisfying 0.60 ≤ ICC < 0.75, and E refers to an excellent category in ICC values satisfying ≥ 0.75. ***Thr*** refers to the proportional threshold.

Regarding Table 7, significant correlations were observed predominantly at lower proportional thresholds (0.15, 0.20), representing highly restricted, sparse network states where only the strongest functional connections are retained in the HC‐boys cohort (*n* = 32). As detailed in the table, subclinical variations in the Turgay Conduct Disorder, Hyperactivity, and Inattention scales demonstrated a robust positive correlation with localized network segregation metrics, specifically CC and LE, within the slow‐wave delta and fullband networks. Conversely, these clinical dimensions were strongly and negatively correlated with network assortativity (*R*_as_) in the faster alpha3 and beta bands. Furthermore, Stroop Block 3–5 self‐corrections exhibited a highly specific and reliable spectral divergence driven by these discriminative metrics. The behavior correlated negatively with localized segregation CC and LE in the theta2, alpha, and alpha1 bands and with the *R*_cc_ in the alpha2 and beta bands. GE exhibited only selective associations, validating its more restricted topological role. For the HC‐girls cohort (*n* = 10), the correlation patterns revealed a remarkably higher mathematical determinism, characterized by absolute correlation coefficients approaching unity (*ρ* ≥ 0.96, p ≤ 0.04). As shown in Table 7, Stroop Block 2–5 Completion Time was negatively correlated with GE, CC, and LE in the alpha2 band but positively correlated with GE in the delta/fullband networks. NSS scores, such as Total Gait and Station, showed a selective negative correlation with GE in the theta/theta1 bands and with CC/LE in the alpha2 band.

The correlation profile underwent a significant topological and spectral shift in the ADHD cohorts, as documented in Table 8. In ADHD‐boys (*n* = 30), clinical symptoms bypassed the slow‐wave configurations seen in HCs; instead, Turgay Inattention scores correlated positively and directly with GE, *T*_tran_, and *R*_as_ within the alpha2 band at sparse‐to‐moderate thresholds (0.15, 0.20). Beyond these executive‐symptom associations, NSS Total Gait and Station scores were also positively correlated with *R*_cc_ (ρ = 0.52, p = 0.006) across the alpha3 and beta bands, spanning moderate‐to‐dense thresholds (0.20, 0.25, and 0.30), and with *T*_tran_ (ρ = 0.40, p = 0.045) in the alpha3 band at relatively dense network (Thr = 0.25). Unlike the sparse‐threshold executive correlations, this motor‐behavioral coupling in ADHD‐boys therefore extends into denser network configurations as well, indicating that the sparse‐versus‐dense distinction between boys and girls is not absolute but domain‐dependent (executive vs. motor). In sharp contrast, the ADHD‐girls cohort (*n* = 10) demonstrated a highly distinct topological phenomenon where meaningful clinical correlations emerged almost exclusively at higher proportional thresholds (0.25, 0.30), which accommodate more connections, establishing a dense network topology. As outlined in Table 9, Stroop Block 3–5 self‐corrections were profoundly and negatively correlated with GE, LE, and *R*_as_ at the highest proportional threshold (0.30), meaning a dense network, across the alpha3, beta, delta, and fullband. Similarly, Turgay conduct disorder symptoms in ADHD‐girls were positively linked to LE in the alpha3 band strictly at a dense network (0.30 threshold). NSS Total Gait and Station scores also demonstrated strong positive correlations with GE, *R*_as_, *R*_cc_, and *Q*_mod_ predominantly at these inclusive and dense thresholds (0.25, 0.30).

**TABLE 9:** Summary of cross‐band common directed edges and corresponding effect sizes across group comparison scenarios based on averaged thresholded connectivity matrices by DTF in 4 s.

	*edge*	Effect size		
		*theta*	*alpha3*	*beta*
HC‐boys vs. HC‐girls	**FC6‐T8**	**0.18**	**0.22**	**0.17**
	**FC6‐F8**		**0.14**	**0.14**
	O1‐FC5		0.10	0.10
	F8‐FC5		0.06	0.06
ADHD‐boys vs. ADHD‐girls	**AF3‐T7**	**0.14**	**0.12**	**0.16**
HC‐boys vs. ADHD‐boys	**FC6‐T8**	**0.19**	**0.18**	
	**F4‐F8**		**0.14**	**0.12**
	AF4‐T8		0.09	0.10
	T7‐F4		0.05	0.06
HC‐girls vs. ADHD‐girls	AF3‐O1	0.09		0.12
	T8‐O1	0.06	0.14	
	F3‐AF3		0.09	0.08
	F3‐O1		0.09	0.09

*Note*: The listed electrode pairs provided statistically significant directional connectivity alterations (*p* < 0.05). Bold values refer the larger effect sizes.

Across all analyses, several graph metrics emerged repeatedly as the most stable discriminative markers. LE, CC, *T*_tran_, *R*_cc_, and *Q*_mod_ consistently differentiated either healthy girls from healthy boys, ADHD‐girls from ADHD‐boys, or ADHD‐girls from HC‐girls. In contrast, GE exhibited more selective alterations that were largely confined to alpha‐related frequency bands and specific connectivity estimators. Based on these findings, the primary correlation analyses should focus on the graph metrics that repeatedly demonstrated reproducible group differences, particularly LE, CC, *T*_tran_, *R*_cc_, and *Q*_mod_. These indices are expected to provide the greatest sensitivity for detecting associations with behavioral performance because they consistently represented the strongest and most reliable network alterations throughout both the within‐group and between‐group comparisons.

To evaluate directional information flow across brain regions, DTF was computed using 4‐s epoch windows (W_4_sec) across multiple frequency bands, specifically theta, alpha3, and beta. In line with prevailing neurophysiological literature, sparse network topologies were initially characterized using conservative threshold densities of 0.15 and 0.20. Consequently, our edge‐by‐edge connectivity analysis focused on these specific cross‐threshold combinations where statistically significant differences were stabilized. To determine localized connectivity alterations, independent‐sample *t*‐tests were performed on an edge‐by‐edge basis across the entire 14 × 14 directional connectivity matrix, excluding self‐connections (diagonal elements). For each directed pair (i.e., source node channel‐i to target node channel‐j), the distribution of DTF values was compared between groups. Statistical significance was established at an uncorrected threshold of *p* < 0.05. To represent the clinical and physiological relevance of these significant connections, an effect size matrix (difference in group means) was mapped exclusively onto the statistically significant edge mask. Topographic visualization of these significant directional flows was achieved using the *circularGraph* MATLAB toolbox, mapping nodes spherically around the circumference to represent directional influx and efflux profiles visually across all cohort comparisons.

When considering Tables 7 and 8 in unison, a striking pattern of cross‐cohort brain‐behavior consistency emerges between the male groups, contrasting with the more volatile and attenuated correlation profiles observed in females. Specifically, both the HC‐boys and ADHD‐boys cohorts exhibited tightly mirrored topological trajectories: the Turgay Oppositional Defiant Disorder (ODD) score correlated positively with CC metrics within the beta band; the Stroop Block 3 Errors score showed direct positive couplings with CC and LE indices within the lower and upper alpha bands, respectively, while additionally projecting positively onto *R*_as_. The Stroop Block 3–5 self‐corrections score correlated positively with GE and *R*_as_ across the theta2, lower alpha, and higher alpha bands; and the Turgay Conduct Disorder score was consistently linked to positive shifts in CC within the delta and fullband spectra. In contrast, the shared cross‐cohort behavior for females (HC‐girls and ADHD‐girls) was notably sparse, confined strictly to a negative relationship between Stroop Block 3–5 self‐corrections and GE across the alpha2/beta bands for the healthy group and the alpha3/beta bands for the clinical group. When evaluated from a health‐versus‐pathology paradigm, broader cross‐gender commonalities became visible. In the healthy cohort (HC‐boys and HC‐girls), the PANESS Total Gait and Station score correlated negatively with GE within the fullband and theta networks, respectively; conversely, within the clinical cohort (ADHD‐boys and ADHD‐girls), this motor index underwent a distinct directional reversal, displaying shared positive correlations with the *R*_cc_ across the alpha3 band. Furthermore, the healthy cohort manifested a synchronized negative correlation between the Turgay Hyperactivity score and *R*_as_ within the alpha2 and alpha3 pipelines. Beyond these highly structured alignments, the remaining behavioral measures failed to yield stable correlation profiles, characterized instead by erratic distributions across cohorts and fluctuating trajectories between slow‐ and fast‐wave oscillations.

**FIGURE 2:**
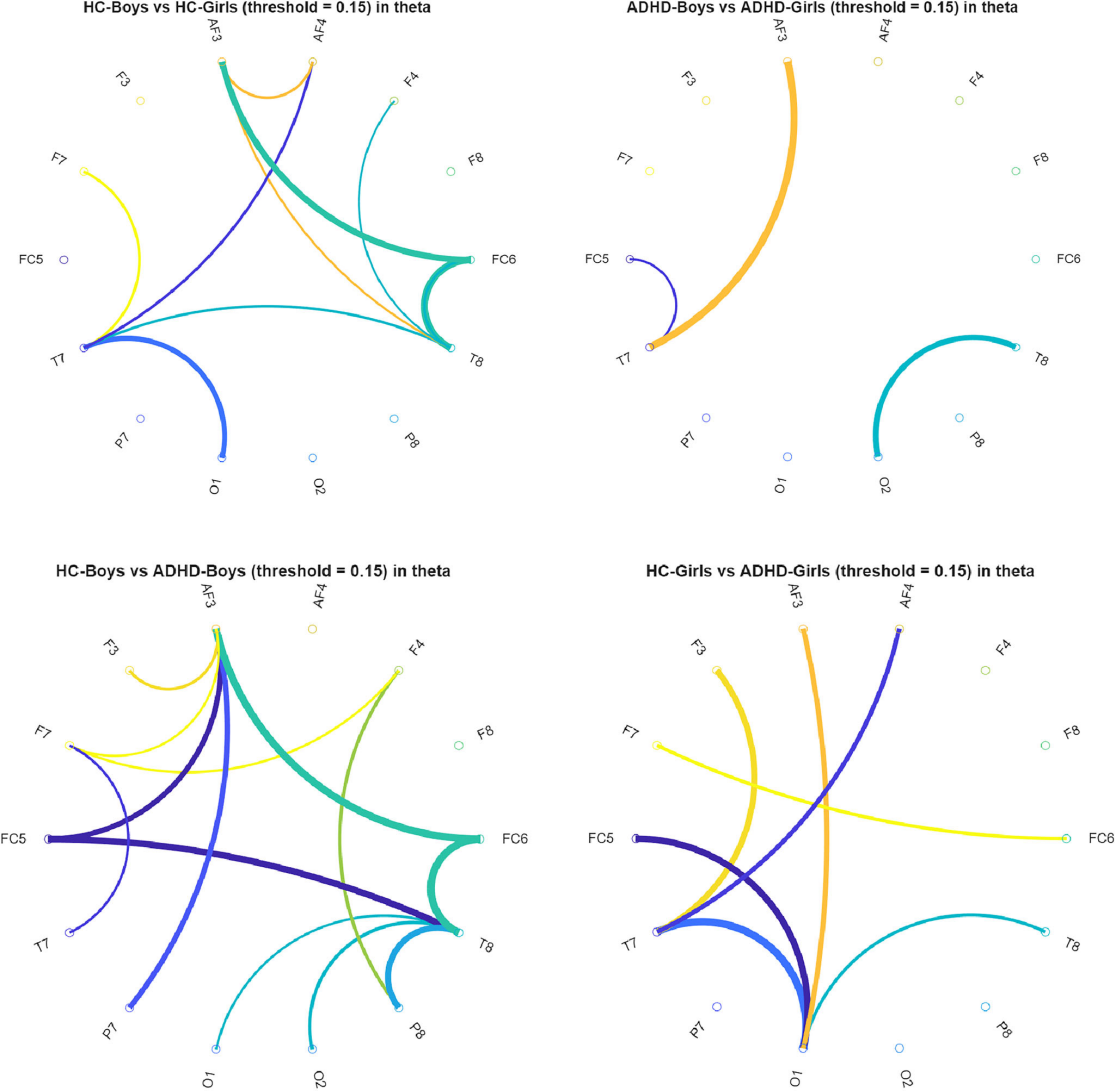
Circular graph visualization of edge‐by‐edge functional connectivity differences derived from DTF matrices within the theta band (4‐s epochs). The diagrams depict statistically significant directional connectivity flows (*p* < 0.05) scaled by effect size across four distinct group comparisons: HC‐Boys versus HC‐Girls, ADHD‐Boys versus ADHD‐Girls, HC‐Boys versus ADHD‐Boys, and HC‐Girls versus ADHD‐Girls.

**FIGURE 3:**
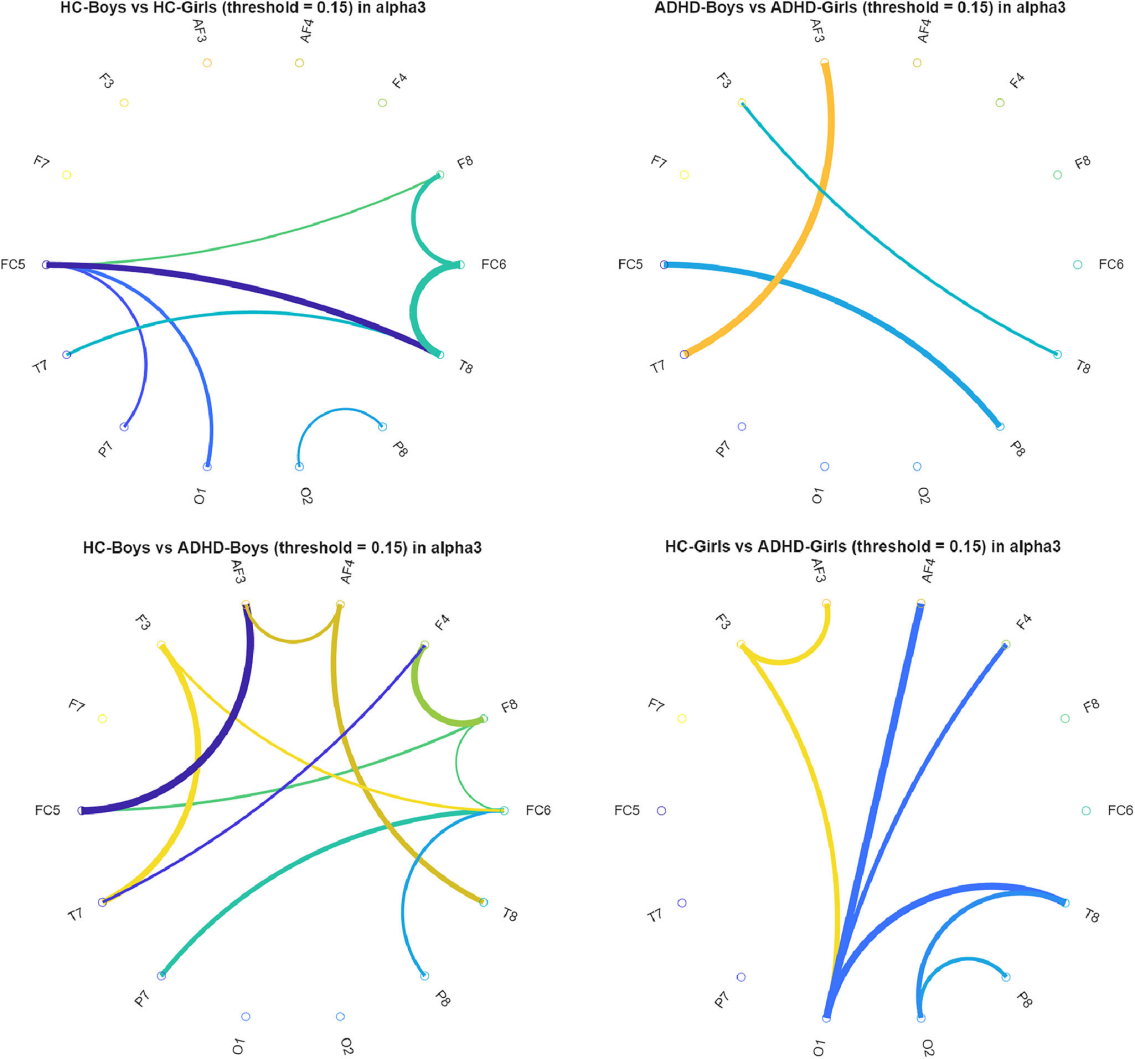
Circular graph visualization of edge‐by‐edge functional connectivity differences derived from DTF matrices within the alpha3 band (4‐s epochs). The diagrams depict statistically significant directional connectivity flows (*p* < 0.05) scaled by effect size across four distinct group comparisons: HC‐Boys versus HC‐Girls, ADHD‐Boys versus ADHD‐Girls, HC‐Boys versus ADHD‐Boys, and HC‐Girls versus ADHD‐Girls.

**FIGURE 4:**
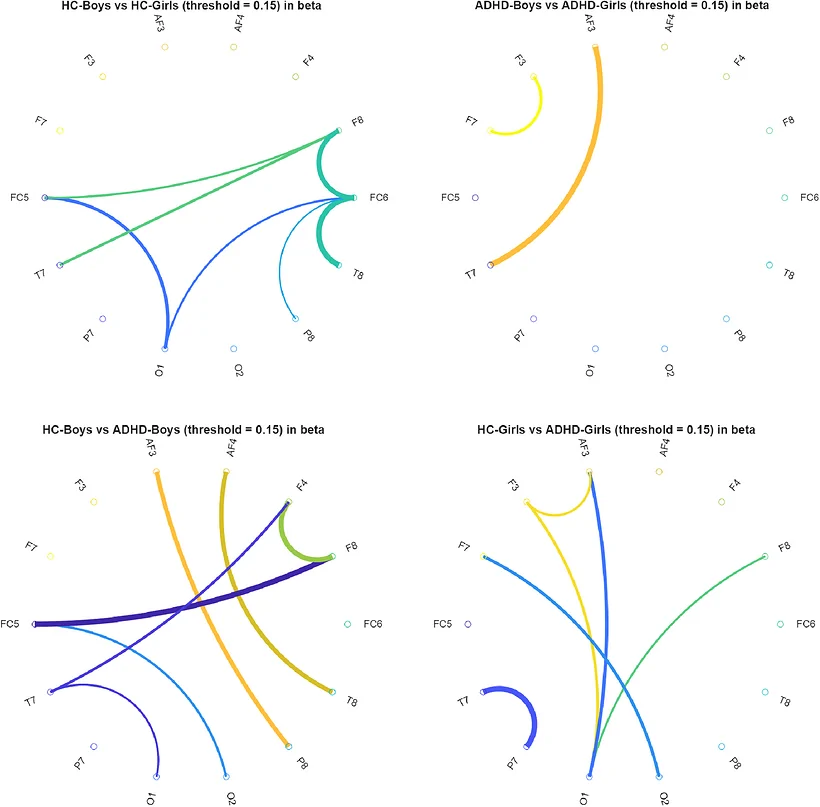
Circular graph visualization of edge‐by‐edge functional connectivity differences derived from DTF matrices within the beta band (4‐s epochs). The diagrams depict statistically significant directional connectivity flows (*p* < 0.05) scaled by effect size across four distinct group comparisons: HC‐Boys versus HC‐Girls, ADHD‐Boys versus ADHD‐Girls, HC‐Boys versus ADHD‐Boys, and HC‐Girls versus ADHD‐Girls.

The topological distribution of these significant directional flows is visually mapped across a series of circular graphs. Specifically, the edge‐by‐edge intergroup connectivity differences derived from the DTF matrices using 4‐s epochs were displayed chronologically in Figure 2 for the theta band, Figure 3 for the alpha3 band, and Figure 4 for the beta band. Each figure comprehensively illustrates the directional influx and efflux profiles across all four group comparison scenarios arranged panel‐by‐panel. Regarding these circular graphs, visual inspection of the thresholded directional connectivity graphs across all three evaluated frequency bands (theta, alpha3, and beta) revealed distinct topological profiles regarding the overall volume of altered pathways. Dynamically, the highest density of statistically significant intergroup connectivity differences (*p* < 0.05) was consistently observed in the male clinical contrast (HC‐Boys vs. ADHD‐Boys). In stark contrast, the internal clinical gender comparison (ADHD‐Boys vs. ADHD‐Girls) systematically exhibited the lowest number of significantly altered directional edges across the spectral pipeline.

A striking topological stability was noted when transitioning to the higher frequency bands; with the sole exception of the intra‐patient gender contrast (ADHD‐Boys vs. ADHD‐Girls), all other group comparisons maintained a highly consistent spatial configuration. Specifically, exactly four distinct electrode pairs demonstrated significantly different connectivity profiles that were structurally preserved across both alpha3 and beta bands. The exact spatial coordinates of these cross‐band common directed edges, along with their respective effect sizes capturing the magnitude of these stable statistical differences, are summarized comprehensively in Table 9. As evidenced by the consolidated empirical data, with the sole exception of the intra‐patient gender contrast (ADHD‐Boys vs. ADHD‐Girls), all remaining cohort comparisons demonstrated exactly four stable directional edges that remained consistently altered either across the entire spectral range (including the theta band) or specifically within the higher alpha3 and beta bands. Among these robustly preserved pathways, the FC6‐T8 directional connection emerged as a highly prominent physiological feature. In the HC gender comparison (HC‐Boys vs. HC‐Girls), this specific edge exhibited significant differences across all three evaluated frequency bands. Furthermore, the same FC6‐T8 pathway demonstrated shared statistical alterations in the higher alpha3 and beta bands during the male clinical contrast (HC‐Boys vs. ADHD‐Boys). Visually and structurally, this connection manifested with a visibly thicker profile in the circular graphs, indicating the highest effect size relative to the other concurrently altered links. Topological patterns distinct to the female clinical cohort revealed that contrasts involving the ADHD‐Girls group waspredominantly characterized by significant connectivity differences converging onto the AF3 locus. These localized shifts were primarily driven by directional influxes originating from the left occipital, left temporal, and left frontal regions. In comparison, the distinct connectivity alterations discovered between healthy and clinical female subjects (HC‐Girls vs. ADHD‐Girls) exhibited a clear spatial distribution that was predominantly dominant within the left frontal and left occipital networks.

A comparative evaluation of clinical alterations reveals a profound topological divergence between genders, indicating that ADHD pathology yields highly distinct neurophysiological network profiles in boys versus girls. The empirical data refutes a uniform pathological signature, demonstrating a clear hemispheric and regional asymmetry. In the female clinical contrast (HC‐Girls vs. ADHD‐Girls), the pathological topography is distinctively centered around posterior cortical nodes, specifically involving the left and right occipital regions (O1 and O2). Across the theta and alpha3 bands, significant anomalies were predominantly manifested in long‐range antero‐posterior pathways (e.g., O1‐AF4, O1‐T8, and F3‐O1). Furthermore, cognitive control networks within the female cohort demonstrate strict spectral preservation in higher frequencies, where the F3‐O1 and F3‐AF3 directed edges remain significantly altered across both alpha3 and beta bands. This posterior‐driven topology suggests that female ADHD manifestations may be robustly linked to alterations in sensory‐visual integration and long‐range attentional allocation networks. Conversely, the male clinical contrast (HC‐Boys vs. ADHD‐Boys) presents a highly dense, localized, and lateralized network disruption. Unlike the occipital‐centric configuration observed in girls, the pathological differences in boys are prominently clustered within the centro‐temporal and frontal frameworks, displaying a marked right‐hemispheric dominance. This is highlighted by the FC6‐T8 pathway, which exhibits the highest statistical effect size in both theta and alpha3 bands, alongside the robust preservation of the right frontal F4‐F8 link in higher bands (alpha3 and beta). Given that right fronto‐striatal and fronto‐temporal circuits are traditionally implicated in motor inhibition and impulse control, this male‐specific right‐hemispheric concentration aligns with the clinically prevalent hyperactive/impulsive phenotypes typically observed in male cohorts, contrasting the more inattentive, sensory‐integration profiles observed in the female clinical network.

## 4 Discussion

To the best of our knowledge, the present study represents the first highly systematic framework evaluating functional brain network architectures in ADHD through multi‐dimensional parameter exploration. Specifically, we comprehensively examined the effects of gender within both healthy and clinical cohorts across multiple methodological dimensions: utilizing directed functional connectivity estimator (DTF) alongside phase‐based PLI variants (wPLI and dwPLI); exploring three distinct temporal windows (1‐s, 2‐s, and 4‐s); mapping seven precise frequency bands, including slow‐wave delta, partitioned theta (theta1 and theta2), segmented alpha (alpha1, alpha2, and alpha3), beta, and the global fullband network; and evaluating network density across five proportional thresholds (0.10–0.30). Because robust, widespread statistical differences emerged between genders in preliminary assessments, the HC and ADHD cohorts were stratified and analyzed independently through dedicated patient‐control comparisons (HC‐girls vs. ADHD‐girls and HC‐boys vs. ADHD‐boys).

Beyond mapping significant topological differences via seven core graph‐theoretical indices, GE indexing network integration, CC and Transitivity (*T*_tran_) reflecting functional segregation and local connectedness, LE capturing local network organization, Modularity (*Q*_mod_) representing network compartmentalization, Assortativity (*R*_as_) quantifying network resilience and mixing patterns, and the *R*_cc_ tracking high‐degree hub hierarchical architectures, the subsequent behavioral analyses were strategically restricted to these high‐sensitivity metrics. This topological battery allowed us to rigorously investigate correlations with NSS, executive functioning profiles from the Stroop Battery, and clinical dimensions from the Turgay DSM‐5‐based scales. Finally, to ground these global trends in localized neural circuitry, we scrutinized the macro‐level edge disruptions between thresholded functional connectivity matrices that were systematically averaged across individual segments. Together, this multi‐threshold and multi‐spectral approach provides a highly granular depiction of functional brain‐behavior dynamics.

Prior to evaluating downstream network topology, a fundamental pillar of this investigation was the rigorous verification of metric reproducibility across the 1050 parameter combinations. Our findings establish that the DTF provides a markedly superior mathematical backbone compared to phase‐sensitive estimators. DTF demonstrated exceptional structural stability, with 53.0% of its combinations achieving excellent reliability (ICC ≥ 0.75) and an additional 29.0% exhibiting good reliability. In stark contrast, phase‐based measures yielded profoundly depressed reliability profiles; PLI yielded only 2.4% excellent combinations, while wPLI and dwPLI fell below 2%. Crucially, roughly 42% of all configurations derived from phase‐based estimators collapsed into the poor reliability category, whereas DTF restricted poor classifications to a negligible 4.4%. This disparity suggests that directed functional connectivity approaches may be more stable. This outcome strongly aligns with recent methodological benchmarks confirming that directed, autoregressive network formulations overcome the inherent volume‐conduction vulnerabilities of non‐directional phase metrics, thereby providing highly reproducible topological landmarks in neurodevelopmental cohorts (Dimitriadis et al. 2023).

Within the typically developing group, sex‐related differences identified by the DTF method exhibited a strict co‐dependence on temporal window length and spectral band. Shorter temporal windows (1 s) demonstrated peak sensitivity for capturing gender dynamics, which progressively narrowed into the alpha sub‐bands as window lengths extended to 4 s. Overall, healthy sex‐related differences were predominantly manifested in network integration (GE), local segregation (LE), and modular segregation (*Q*_mod_), primarily localized within the alpha pipelines. In the 1‐s window, delta alterations were confined to GE and *Q*_mod_ at the sparsest density (0.10), while theta bands revealed shifting modular organization (*Q*_mod_) at moderate thresholds. Moving into the alpha sub‐bands, GE consistently segregated boys and girls at sparse settings, whereas LE and *R*_cc_ emerged exclusively at dense boundaries (0.25–0.30) with high effect sizes. Concurrently, *T*_tran_ captured prominent sex‐dependent variations across alpha2, alpha3, and beta bands.

When examining the phase‐sensitive alternatives, PLI showed the highest sensitivity among phase metrics, reproducing full‐band differences but deviating from DTF by introducing significant alterations in network assortativity (*R*_as_) within the alpha3 and fullband spectra. Furthermore, dwPLI uniquely captured a concurrent reduction in both LE and CC for healthy girls relative to healthy boys within the 1‐s fullband network at moderate thresholds, reinforcing its selective capability to track subtle, localized organizational shifts under highly restricted temporal constraints.

The introduction of ADHD pathology appeared to attenuate the sex differences observed in the healthy group. Within the clinical cohort, the DTF estimator revealed a markedly reduced sensitivity, yielding substantially fewer significant findings that were strictly confined to the 4‐s temporal window. In direct contrast to the healthy cohort's broad threshold distribution, clinical sex differences stabilized exclusively at the sparse boundary (0.15), where LE and CC in the alpha and alpha1 bands exhibited negative effect sizes, confirming higher segregation values in ADHD‐boys than in ADHD‐girls. Conversely, *Q*_mod_ shifted in the opposite direction with positive effect sizes across significant bands.

Phase‐sensitive measures within the ADHD cohort revealed highly discrete, method‐dependent adjustments: dwPLI tracked a frequency‐dependent reversal for GE within the alpha3 (negative) versus beta (positive) bands under identical 2‐s temporal constraints. Furthermore, phase metrics converged at broader thresholds, showing depressed LE and CC profiles for ADHD‐girls in the delta band (Thr = 0.20) alongside an upregulation of modularity (*Q*_mod_) in the theta2 band and GE in the beta network. Collectively, these shifts suggest that ADHD may not simply alter the magnitude of network parameterization but could also reorganize the directional and spatial expression of sex‐related effects.

Due to the profound influence of sex on baseline and pathological connectivity, pooling the sample was rejected to avoid obscuring gender‐specific signatures. In the stratified female contrast (HC‐Girls vs. ADHD‐Girls), the DTF approach revealed a relatively consistent pattern of reduced connectivity. Across all evaluated temporal windows, LE, CC, *T*_tran_, and *R*_cc_ consistently demonstrated severe reductions in ADHD‐girls within the alpha2 band at the dense Thr = 0.30 configuration, yielding exceptionally large negative effect sizes ranging from −1.01 to −1.17. Peak disruptions occurred at the 1‐s window, where LE collapsed across delta, alpha2, alpha3, beta, and fullband networks, while *R*_cc_ tracked a severe down‐regulation of hub hierarchies across the core alpha bands. Conversely, phase estimators (wPLI and dwPLI) yielded a highly restricted, contrasting topology, capturing an isolated increase in modularity (*Q*_mod_) for ADHD‐girls strictly within the theta and theta2 bands at the dense Thr = 0.30 boundary. This divergence suggests that while directed flows capture a widespread disintegration of local information transmission and hub organization, phase‐synchronized components track an increase in macro‐level compartmentalization within slow‐wave oscillations.

The mapping of graph metrics onto behavioral scales suggests a possible neurophysiological distinction between sexes. In the healthy boy cohort, subclinical variations in the Turgay scales directly correlate with localized segregation metrics (CC and LE) within slow delta and fullband networks at sparse thresholds (0.15, 0.20), while correlating negatively with network assortativity (*R*_as_) in faster alpha3 and beta bands. Crucially, as healthy boys engage in cognitive monitoring during Stroop tasks, a highly reliable spectral divergence occurs. Our results demonstrated clear instances of frequency‐dependent reversals, where core graph metrics correlated with the same behavioral score in opposite directions across different frequency bands. Crucially, rather than being driven by global network parameters, this spectral divergence was predominantly mediated by CC, LE, and *R*_cc_ metrics that consistently emerged as the most stable discriminative markers differentiating the cohorts in between‐group comparisons.

A prominent example is found in the HC‐boys cohort for Stroop Block 3–5 self‐corrections (Table 7). This behavioral score correlates positively with CC and LE within slow‐wave configurations (theta2, alpha, and alpha1) but flips to a negative correlation with CC, LE, and *R*_cc_ in the faster alpha2 and beta bands during actual error‐correction execution (Table 8). This frequency‐dependent reversal may point to a dynamic neural strategy:

*The Role of Stable Segregation Metrics (LE and CC)*: In the slow‐to‐medium oscillator channels, the positive correlation indicates that higher localized information processing capacity (LE) and local interconnectedness (CC) directly support the baseline cognitive control required to track ongoing performance and monitor errors.
*The Cortical De‐inhibition Mechanism*: Conversely, the sharp shift to a negative correlation with CC, LE, and *R*_cc_ in the faster alpha2 and beta networks may reflect a more localized release from cortical inhibition. Because LE and CC are highly sensitive discriminative markers for group differences, their negative association with self‐corrections is consistent with the possibility that corrections are achieved not through a generalized global effort, but through a more localized modulation of segregation and rich‐club constraints in faster motor‐sensory rhythms to facilitate rapid information transfer. This framework undergoes a severe spatial and spectral realignment in pathology. In ADHD‐boys, clinical symptoms entirely bypass the slow‐wave configurations seen in HC; instead, Turgay Inattention scores correlate positively and directly with GE, transitivity (*T*_tran_), and assortativity (*R*_as_) strictly within the alpha2 network at sparse‐to‐moderate thresholds. In sharp contrast, the ADHD‐girls cohort exhibits a distinct topological phenomenon: meaningful behavioral correlations emerge almost exclusively at higher proportional thresholds (0.25, 0.30), which establish a dense network topology. At strict, sparse backbone levels, secondary functional connections are pruned, yet. These findings are compatible with a compensatory pattern that may mask underlying neural differences.

However, when cognitive demands or clinical severity force the recruitment of broader, peripheral sub‐circuits at dense thresholds, this apparent compensation may no longer hold. Consequently, the underlying pathology becomes visible through exceptionally strong correlation coefficients. This breakthrough is marked by a profound multispectral collapse, where Stroop self‐corrections correlate negatively with GE, LE, and *R*_as_ across the alpha3, beta, delta, and fullband networks, and an adaptive escalation where Turgay Conduct dimensions (LE in alpha3) and NSS Gait/Station scores (GE, *R*_as_, *R*_cc_, and *Q*_mod_) project heavily onto these dense architectural configurations. These outcomes are broadly consistent with recent clinical neuroimaging work suggesting that females with neurodevelopmental conditions exhibit a robust “neural reserve” or compensatory shielding that effectively maintains core modular backbones under low‐demand states, only breaking down under diffuse, high‐density network configurations (Sciberras et al. 2022; Marino and Mantini 2026). By focusing the correlation narrative on these high‐sensitivity metrics (LE, CC, *R*_cc_, *T*_tran_, and *Q*_mod_), we ensure that the brain‐behavior associations reported are directly linked to the most reliable network alterations characterizing ADHD and gender typologies.

When grounding these macro‐topological trends into localized neural pathways via edge‐by‐edge DTF analysis, a striking regional and hemispheric asymmetry is revealed between genders. Visualized across Figures 2, 3, 4, the highest density of statistically significant intergroup connectivity differences is systematically concentrated within the male clinical contrast (HC‐Boys vs. ADHD‐Boys), whereas the internal gender contrast (ADHD‐Boys vs. ADHD‐Girls) presents the sparsest edge profile. With the sole exception of the intra‐patient gender comparison, all cohort scenarios maintain a remarkably stable spatial configuration, preserving exactly four distinct electrode pairs across both the alpha3 and beta pipelines.

Among these stable pathways, the FC6‐T8 directional connection represents a major physiological marker. This specific edge is significantly altered across all three evaluated frequency bands in healthy gender contrasts and remains structurally preserved with the highest statistical effect size (manifesting as a visibly thicker profile) across the alpha3 and beta bands in the male clinical contrast. Because right fronto‐striatal and fronto‐temporal circuits are structurally charged with motor inhibition, executive tracking, and impulse control, this pattern may offer a plausible neural correlate of the hyperactive/impulsive phenotypes prevalent in male cohorts. This right‐lateralized frontal and frontotemporal is broadly consistent with the Right Hemisphere Hypothesis, which implicates these specific circuits in motor inhibition, impulsivity, and sustained attention deficits (Faraone et al. 2021; Rubia 2022).

Conversely, the network abnormalities mapped in the female clinical contrast (HC‐Girls vs. ADHD‐Girls) completely bypass this localized right‐hemispheric configuration, shifting instead to long‐range antero‐posterior networks anchored around left frontal and posterior occipital (O1, O2) nodes. Pathological differences in the female cohort are notably characterized by significant directional influxes converging onto the AF3 locus from left occipital and temporal regions, alongside cross‐band stable alterations in the F3‐O1 and F3‐AF3 pathways. In clinical literature, ADHD in girls is predominantly characterized by the inattentive subtype and sensory‐gating difficulties rather than overt hyperactive‐impulsive behaviors (Biederman et al. 2002; Rucklidge 2010). The occipital‐centric and long‐range prefrontal‐posterior network disorganization captured here may offer a neurophysiological correlate *for these variances, which* could help explain why their underlying deficits remain less apparent at sparse thresholds but break through during widespread network recruitment.

The present findings are consistent with previous studies reporting altered functional connectivity and atypical network organization in ADHD (Konrad and Eickhoff 2010; Lin et al. 2014; Beare et al. 2017). In particular, the observed pattern characterized by reduced efficiency and increased modularity is consistent with prior evidence suggesting a shift toward less integrated and more segregated network configurations in ADHD. The findings also extend previous EEG studies (Clarke et al. 2001, 2003) by indicating that sex‐related differences in ADHD may involve not only spectral characteristics but also differences in network topology and brain–behavior relationships.

Importantly, the present study demonstrates associations between NSS and EEG‐derived network metrics in children with ADHD. Rather than reflecting isolated motor abnormalities alone, these findings support the interpretation that NSS may represent behavioral manifestations of altered sensorimotor network integration and atypical maturation of distributed neural systems. This interpretation is consistent with studies suggesting that NSS are associated with abnormalities in distributed cortical, subcortical, and cerebellar circuits (Mostofsky et al. 2003; Mostofsky et al. 2006; Mostofsky et al. 2007). The observed associations between motor measures and graph‐theoretical metrics further support the relevance of examining motor and executive domains within a shared network‐based framework.

## 5 Conclusion

The present study demonstrates that graph‐theoretical analysis of resting‐state EEG connectivity can provide quantitative information regarding functional brain network organization in children with ADHD. The findings indicate that alterations in network integration and segregation are associated with both executive dysfunction and NSS.

The observed relationships between graph‐theoretical metrics and behavioral measures suggest that executive and sensorimotor difficulties in ADHD may reflect related alterations in large‐scale functional network organization rather than entirely independent symptom domains. In particular, the associations between NSS and network topology support the interpretation that motor abnormalities in ADHD may be linked to atypical sensorimotor network maturation and impaired integration across distributed neural systems.

The findings also indicate that brain–behavior relationships differ between boys and girls, suggesting sex‐dependent variability in the organization of functional brain networks in ADHD. These results extend previous connectivity findings by integrating executive performance, motor measures, and network‐level EEG metrics within a single framework.

Although further longitudinal and multimodal studies are required to clarify developmental mechanisms and causal relationships, the present findings support the value of combining EEG‐based connectivity analysis with graph‐theoretical approaches to investigate the neurophysiological organization underlying behavioral variability in ADHD.

Our study also demonstrates that the functional brain network correlates of ADHD and healthy subclinical variations are deeply governed by gender‐specific topodynamics and network density constraints. Crucially, brain‐behavior associations are predominantly mediated by localized segregation and hub parameters (LE, CC, *T*_tran_, *R*_cc_, and *Q*_mod_), which consistently represent the most stable and reproducible discriminative markers across cohorts. While healthy cohorts balance cognitive demands through automated, sparse core configurations isolated at lower thresholds (sparse networks), ADHD pathology forces a reliance on fast alpha and beta sensory‐motor rhythms through a high‐effort recruitment of denser network topologies. Most notably, the clinical phenotype of ADHD in female patients appears to spare the automated sparse core backbone, with significant behavioral correlations emerging exclusively when the network transitions into a highly integrated, dense architecture at higher thresholds (0.25, 0.30). This phenomenon provides a plausible neurophysiological substrate for the diagnostic camouflage effect observed in females. These findings emphasize the clinical necessity of utilizing multi‐threshold graph theoretical frameworks and gender‐related designs, focusing on high‐sensitivity discriminative metrics to fully decode the neurobiological landscape of ADHD.

In addition, our edge‐level directional connectivity analysis demonstrates that ADHD does not possess a uniform neurophysiological signature; rather, it manifests *through* highly gender‐specific circuit disruptions, characterized by a prominent right‐hemispheric centro‐temporal deficit in boys versus an antero‐posterior occipital‐centric network disorganization in girls, underscoring the critical need for gender‐tailored diagnostic and therapeutic frameworks in clinical neurophysiology.

## 6 Limitations

Formal standardized IQ testing was not performed. Consequently, subtle differences in cognitive ability may not be completely excluded. The relatively small number of girls (10 in HC, 10 in ADHD) limited the statistical power for detecting sex‐specific effects. Therefore, the observed sex‐related differences should be considered preliminary and require replication in larger, more balanced pediatric ADHD cohorts.

## Author Contributions

**Serap Aydın**: conceptualization, investigation, methodology, visualization, Writing – review and editing, software, data curation, project administration, formal analysis. **Serhat Türkoğlu**: resources, supervision, data curation, funding acquisition. **Merve Kuz**: conceptualization, investigation, funding acquisition, writing – original draft, data curation, formal analysis. **Halit Necmi Uçar**: conceptualization, investigation, funding acquisition, data curation, supervision, resources. **Fatih Hilmi Çetin**: conceptualization, investigation, supervision, resources, project administration.

## Ethics Statement

These structured dialogues with both children and parents culminated in a consensus diagnosis by experienced child psychiatrists. Reflecting a commitment to ethical rigor, the study received formal approval from the Selçuk University Medical Faculty Hospital Ethics Committee (Protocol No: 2021/477). Informed consent was obtained from the patients' parents.

## Conflicts of Interest

The authors declare no conflicts of interest.

## Data Availability

The data that support the findings of this study are available from the first author upon reasonable request.
